# Microbial Pathways for Cost-Effective Low-Carbon Renewable Indigoidine

**DOI:** 10.1021/acssuschemeng.4c09962

**Published:** 2025-02-14

**Authors:** Nawa Raj Baral, Deepanwita Banerjee, Thomas Eng, Blake A. Simmons, Aindrila Mukhopadhyay, Corinne D. Scown

**Affiliations:** a Joint BioEnergy Institute, Lawrence Berkeley National Laboratory, Berkeley, California 94720, United States; b Biological Systems and Engineering Division, Lawrence Berkeley National Laboratory, Berkeley, California 94720, United States; c Energy Analysis and Environmental Impacts Division, Lawrence Berkeley National Laboratory, Berkeley, California 94720, United States; d Energy & Biosciences Institute, University of California, Berkeley, California 94720, United States

**Keywords:** biomass sorghum, sugar utilization, aromatics utilization, titer and yield, indigoidine, microbial pathway optimization

## Abstract

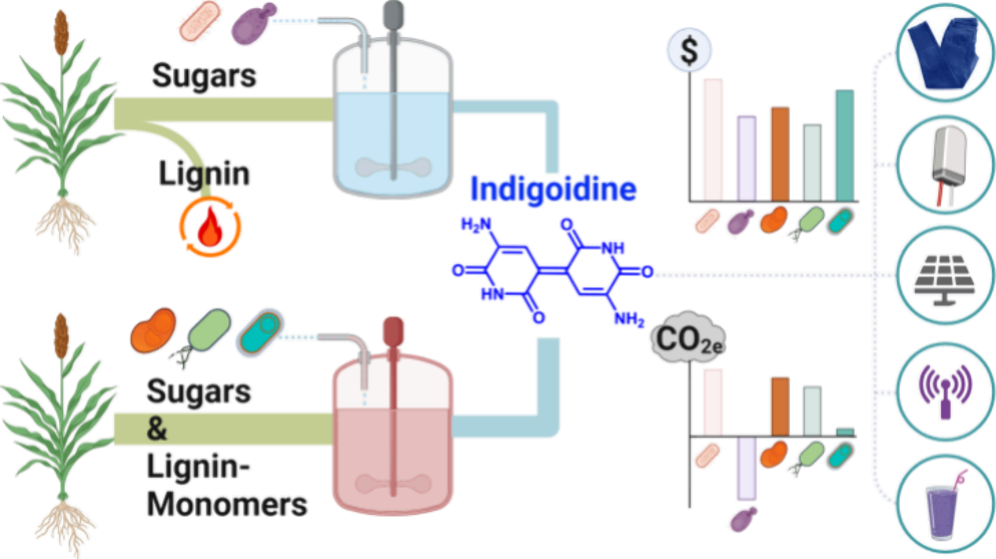

Indigoidine is a bioadvantaged platform molecule with diverse applications, including use as a textile dye, biotransistor, biosolar cell, biosensor, and food coloring. There are multiple microbial hosts and carbon sources that can be used and optimized for its production, yet there is limited guidance for which options have the greatest commercial potential. Here, we consider five different host microbes and combine genome-scale metabolic models with techno-economic and lifecycle assessment models. *Pseudomonas putida* currently outperforms synthetic indigo production and other indigoidine-producing hosts, using glucose, xylose, and lignin-derived aromatics to produce indigoidine at a minimum selling price of $2.9/kg and a greenhouse gas (GHG) footprint of 3.5 kgCO_2e_/kg. Optimizing pathways—achieving 90% of the theoretical indigoidine yield from sugars and aromatics—can reduce costs 6–7-fold and GHG emissions 3–10-fold. From a cost perspective, microbes that co-utilize aromatics are advantageous, while selecting hosts that coproduce other value-added molecules can reduce GHG emissions. System-wide improvements and the use of a low-cost, low-carbon nitrogen source are crucial for commercial viability in all cases.

## Abstract 2

Renewable indigoidine offers a cost-effective, low-carbon alternative to synthetic indigo dye, with potential for other applications.

## 1 Introduction

**Figure 1:**
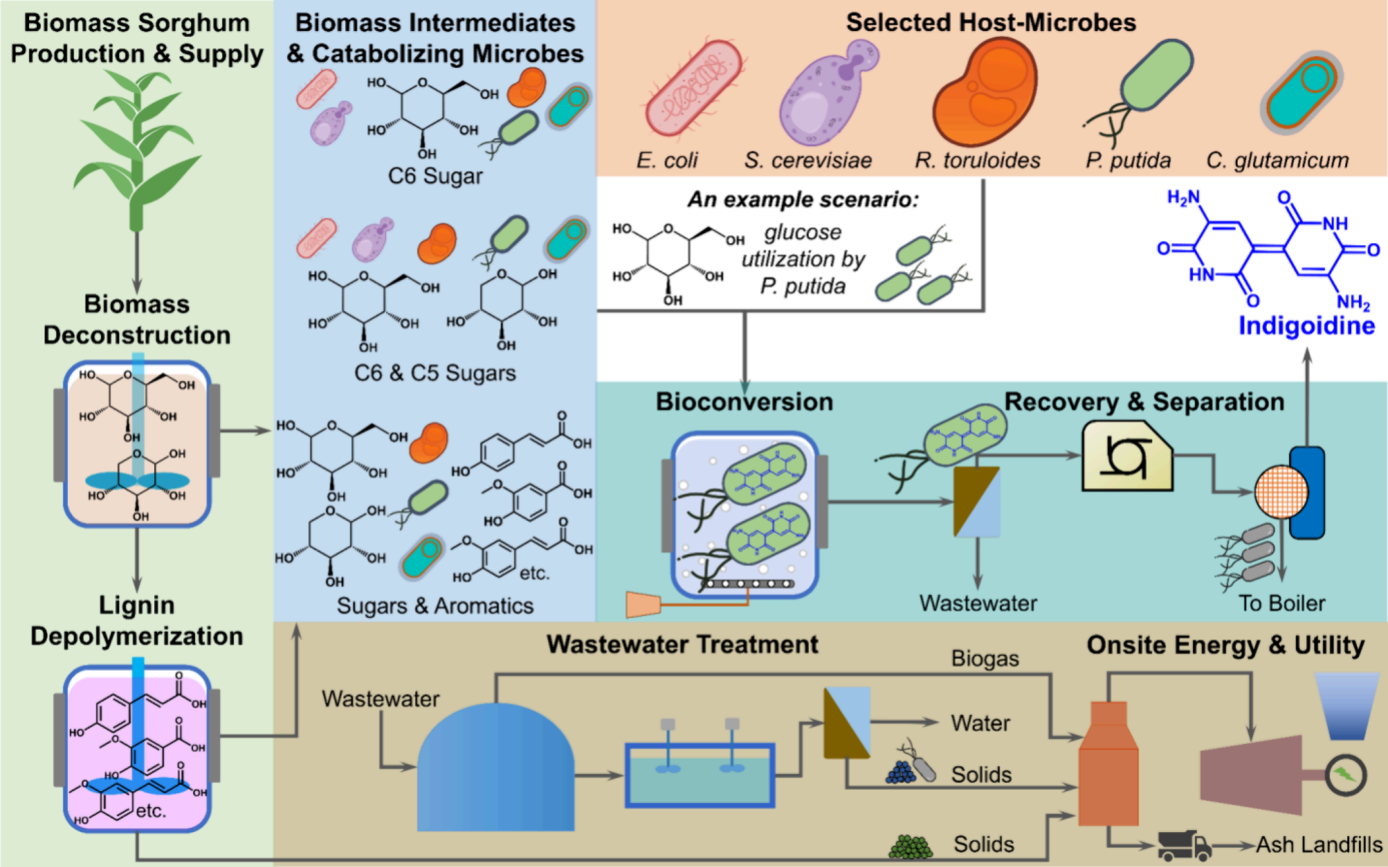
Overview of the biomass sorghum-to-indigoidine production process with options for biomass-derived intermediates and host microbes.

Renewable indigoidine is a prime example of a bioadvantaged platform molecule with several high-value potential applications. It is a natural blue pigment with the potential to replace synthetic indigo dye in fabric dyeing due to its similar color properties, including color strength and dye fixation rate, which measures the percentage of dye that adheres to the fabric.^1^ Apart from industrial dyeing applications, indigoidine is safe to use for food coloring due to its antioxidant and antimicrobial properties.^2^ Additionally, indigoidine could be used to produce biotransistors, biosolar cells, and biosensors due to its conjugated aromatic moiety and intermolecular hydrogen bonding (Figure 1).^3^ Considering only the dyeing application, the global market for renewable indigoidine could be similar to that of synthetic indigo dye, at approximately 80 to 110,000 metric tons per year,^4,5^ generating about 0.37 to 1.52 million metric tons of CO_2e_ per year (Supporting Information (SI),Table S1). About 95%^6^ of this dye is used for dyeing denim garments, and its demand has been increasing at a consistent average growth rate of about 5.1% per year.^7^ Other potential uses of renewable indigoidine are in the exploratory phases, but published studies indicate its promise.^2,3^ Existing biobased alternatives, such as natural indigo dye, directly extracted from *Indigofera tinctoria*, are too costly and land-intensive.^8,9^ The indigo plant yields only 32–326 kg of dye annually per hectare of land and results in production cost averaging of $228/kg (ranging from $15 to $1058 per kg indigo)^8,10^ as compared to synthetic indigo, which costs $5 to $9.1 per kg.^8,10,11^ This study explores the potential for microbially produced indigoidine to provide a lower-cost, lower-greenhouse gas (GHG) alternative to indigo and a wide variety of other products.

Prior experimental studies have investigated indigoidine production in various microbial hosts, including *Escherichia coli*,^12,13^*Saccharomyces cerevisiae*,^14^*Rhodosporidium toruloides*,^15^*Pseudomonas putida*,^16−19^ and *Corynebacterium glutamicum*.^1^ However, there has been no systematic comparison of the options for hosts, metabolic pathways, and carbon sources to better understand microbially produced indigoidine’s long-term potential and prioritize among the available options. By constructing a genome-scale metabolic model for biologically produced renewable indigoidine and combining it with techno-economic analysis (TEA) and life cycle assessment (LCA) modeling, this study focuses on identifying the most promising approaches from a cost and GHG standpoint.

The biological production process for renewable indigoidine can be carried out using sugars and/or bioavailable aromatic monomers. These molecules can be sourced from a wide variety of feedstocks, including sugar, starch, and lignocellulosic materials. The microbial hosts previously used for indigoidine production mostly utilized glucose as a carbon source, while a few studies utilized l-glutamine,^12,13^ aromatics,^18^ xylose,^19^ and lignocellulosic biomass^15,17^ hydrolysate. The experimental titers, rates, and yields ranged from 0.98 to 49.3 g/L, 0.01 to 0.96 g/L/h, and 4.5 to 33 wt %, respectively. The highest reported titer and rate were achieved using glucose in *C. glutamicum* with soy sauce-enriched media, while the highest indigoidine yield was achieved using glucose in *P. putida* with minimal medium. *P. putida* also efficiently utilizes aromatics, which can be derived from the lignin fraction of biomass.^15^ To date, the *P. putida* has been shown to produce indigoidine using *para*-coumarate in a minimal medium at a titer, rate, and yield of 7.3 g/L, 0.15 g/L/h, and 74 wt %, respectively (77% of the maximum theoretical yield).^17^*R. toruloides*, which is known for its ability to use a diversity of carbon sources, achieved an indigoidine yield of 4.5 wt % from a lignocellulosic biomass-derived hydrolysate (not subjected to lignin depolymerization).^15^ In addition to indigoidine, some of these host organisms naturally produce coproducts. For example, the eukaryotic fungus *S. cerevisiae* produces ethanol and the prokaryotic bacterium *C. glutamicum* produces lactic acid and some amino acids along with indigoidine (Figure 1).

The experimental results published to date demonstrate both a diversity of options and meaningful improvements in the titer, rate, and yield of indigoidine production. However, how these options compare at a system level, and the degree to which any of them would have to be further optimized at scale, is not well understood. A recent economic analysis focused specifically on the bioconversion and recovery processes^20^ evaluated the impacts of indigoidine production rate on net present value, payback period, and minimum selling price using *C. glutamicum* and glucose as the feedstock. The limited scope of existing literature underscores the need for a comprehensive TEA and LCA across multiple hosts and pathways, which are crucial for prioritizing future research and development, as well as accelerating the commercialization of the technology.

For the purposes of this study, we model the deconstruction of lignocellulosic biomass to sugar and aromatic monomers, which are then catabolized by host microbes to produce indigoidine. We design and simulate five different systems that use *E. coli*, *S. cerevisiae*, *R. toruloides*, *P. putida*, and *C. glutamicum*, to convert the resulting sorghum biomass-derived hydrolysate and demonstrate systematic comparison of the options for hosts, metabolic pathways, and carbon sources. This comparison is made by comprehensively assessing the capital and operating costs, minimum selling price, and life cycle GHG footprint of renewable indigoidine at the current state of the technology and potential future improvements. These economic and environmental metrics are documented for each stage of the entire field-to-indigoidine production chain. Furthermore, we explore the optimal indigoidine and coproduct mix, the most influential process parameters, process bottlenecks, and improvement opportunities. These results can inform decisions within scientific research communities, companies seeking to scale indigoidine production, and the broader community of researchers and private industry working on microbially produced bioproducts.

## 2 Methods

### 2.1 Modeling Overview

We developed separate process models for the selected host microbes, including *E. coli*, *S. cerevisiae*, *R. toruloides*, *P. putida*, and *C. glutamicum*, all with a biorefinery size of 2000 bone-dry metric tons of biomass sorghum intake per day (Figure 1). This scale is consistent with a typical *n*th plant analysis. However, given the comparatively smaller size of the indigo market relative to commodity chemicals and fuels, it is possible that smaller scales may be viable and this warrants further analysis. Additionally, further testing of indigoidine’s properties as a replacement for synthetic indigo dye can ensure that the volume of indigoidine needed to replace a functionally equivalent volume of synthetic indigo is adjusted as needed (Figure S1).

Biomass sorghum is selected as a representative feedstock for our modeling due to its advantages, including compatibility with current bioenergy frameworks, an existing forage market, high carbohydrate content, high yield, and tolerance to drought, disease, and heat.^21^ It also has high nitrogen and water use efficiencies.^21^ The results based on sorghum are expected to be generally representative of other potential lignocellulosic feedstocks. The upstream biomass production and supply process, biomass handling and preprocessing, and biomass deconstruction processes remain the same for all the selected microbial hosts; this is described in subsequent sections. The primary process modeling differences among the chosen host microbes lie in the bioconversion process and whether lignin depolymerization is required. The differences in the bioconversion stage depend on which carbon sources (e.g., sugars and aromatics) the host utilizes, which coproducts it generates in addition to indigoidine, the oxygen needed for cellular redox balance, and the nitrogen sources essential for microbial function. Lignin depolymerization is included for *R. toruloides*, *P. putida*, and *C. glutamicum* because these hosts can catabolize aromatics, while *E. coli* and *S. cerevisiae* do not. Variations in deconstruction and bioconversion impact downstream processes, such as indigoidine recovery and separation, wastewater treatment, and onsite energy and utility stages. Each scenario captures these impacts and the resulting changes to material, energy, and equipment costs. The following sections discuss unit processes and data sources for the entire field-to-indigoidine production chain. The major data inputs for different stages are summarized in Table 1, and further details are documented in the Tables S2 and S3.

**Table 1:** Major Inputs Used to Develop the Field-to-Indigoidine Process Model in This Study

**parameter**	**unit**	**current yield**	**baseline yield**^a^	**optimal yield**^a^
biorefinery size^a^	bdt/day	2000	2000	2000
biomass sorghum feedstock cost^21^	$/bdt	118.17	118.17	87.48
biomass sorghum GHG footprint^21^	kgCO_2e_/bdt	144.75	144.75	95.64
soil organic carbon sequestration^29^	kgCO_2e_/bdt	46.00	46.00	77.69
**Biomass sorghum composition**(25,30)				
cellulose	wt %	35.40	35.40	40.00
hemicellulose	wt %	20.70	20.70	29.79
lignin	wt %	21.00	21.00	9.89
**Biomass deconstruction**(25)				
solid loading for pretreatment	wt %	30.00	30.00	40.00
ionic liquid (IL) loading	wt %	5.00	5.00	5.00
IL recovery	wt %	95.00	95.00	98.00
enzyme loading	mg/g-glucan	29.41	29.41	10.00
initial solid loading for hydrolysis	wt %	20.00	20.00	25.00
cellulose to glucose	wt %	75.86	75.86	95.00
xylan to xylose	wt %	60.76	60.76	90.00
**Lignin depolymerization**(27)				
NaOH loading rate	%	2.00	2.00	2.00
NaOH cost	$/kg	0.53	0.53	0.53
lignin to lignin monomer	%	26.70	26.70	50.00
**Bioconversion**				
Indigoidine production in *E. coli*(12)^b^				
ammonium sulfate loading	g/L	26.20	34.2	102.50
air supply	m^3^/s	9.20	16.98	42.50
bioconversion time	h	72.00	72.00	48.00
glucose-to-indigoidine	wt %	8.26	27.55	49.60
xylose-to-indigoidine	wt %	0.00	27.27	49.10
*Indigoidine production inS. cerevisiae*(14)^b^				
ammonium sulfate loading	g/L	26.70	27.01	64.75
air supply	m^3^/s	8.00	8.86	16.46
bioconversion time	h	72.00	72.00	48.00
glucose-to-indigoidine	wt %	4.90	17.22	30.99
xylose-to-indigoidine	wt %	0.00	12.40	22.32
*Indigoidine production inR. toruloides*(15)^b^				
ammonium sulfate loading	g/L	29.9	42.5	133.88
air supply	m^3^/s	9.50	19.05	39.46
bioconversion time	h	120.00	120.00	48.00
glucose-to-indigoidine	wt %	9.31	33.75	60.76
xylose-to-indigoidine	wt %	0.00	33.89	61.00
lignin monomers-to-indigoidine	wt %	0.00	44.88	80.78
*Indigoidine production inP. putida*(16,19)^b^				
ammonium sulfate loading	g/L	37.68	46.8	142.60
air supply	m^3^/s	11.60	19.03	35.89
bioconversion time	h	116.00	116.00	48.00
glucose-to-indigoidine	wt %	33.00	35.13	63.24
xylose-to-indigoidine	wt %	32.00	37.20	66.96
lignin monomers-to-indigoidine	wt %	73.91	49.44	88.99
*Indigoidine production inC. glutamicum*(1)^b^				
ammonium sulfate loading	g/L	26.27	26.34	62.20
air supply	m^3^/s	8.17	12.97	19.60
bioconversion time	h	51.00	51.00	48.00
glucose-to-indigoidine	wt %	14.00	13.78	24.79
xylose-to-indigoidine	wt %	0.00	13.22	23.81
lignin monomers-to-indigoidine	wt %	0.00	39.55	71.19

a Assumed for analysis in this work.

b Calculated in this study using genome-scale metabolic models (Section S6).

### 2.2 Biomass Sorghum Production and Supply

The biomass sorghum production and supply portion of the model includes sorghum cultivation, harvesting, transportation, and storage, which has been adapted from our previous work.^21^ The modeling methods and major assumptions are briefly discussed in Section S2, and more detailed discussion is documented in our prior work.^21^ Major assumptions for biomass sorghum production and supply include an average biomass sorghum yield of 17.9 bone-dry metric tons per hectare (∼8 bone-dry short tons per acre),^21,22^ a uniformly distributed 5% cultivation of biomass sorghum in the entire biorefinery (which impacts the transportation distances modeled),^21^ and a total dry matter loss of 11.6%^21^ across the entire supply chain. The moisture content of the biomass bales delivered at the biorefinery gate is assumed to be 20%.^21^

### 2.3 Biomass Preprocessing

Biomass preprocessing involves biomass handling, milling, and short-term storage at the biorefinery. Biomass bales are conveyed from the storage unit to the shredder and then to the hammer mill to reduce the particle size, typically within the range of 0.9–1.5 cm for biomass pretreatment, although the optimal particle size for efficient biomass deconstruction remains the subject of research.^23^ The milled biomass is temporarily stored in a silo. A belt scale is utilized to measure the required quantity of biomass for pretreatment, which is then delivered to the pretreatment reactor throat. Information regarding process equipment, as well as the necessary capital and operating resources, was collected from prior studies.^23,24^

### 2.4 Biomass Deconstruction

Biomass deconstruction includes five distinct unit operations: pretreatment, neutralization, enzymatic hydrolysis, solid (primarily lignin) separation, and ionic liquid (IL) recovery. We consider the use of a biocompatible IL, cholinium lysinate ([Ch][Lys]), for biomass pretreatment, which enables pretreatment, neutralization, and hydrolysis without the need for a separation step, eliminating the requirement for washing the pretreated biomass with water.^25^ Pretreatment is conducted at an IL loading rate of 5 wt % at 140 °C for 3 h (Table 1 and Table S3). Following pretreatment, sulfuric acid is introduced to neutralize the slurry before it is sent to the hydrolysis reactor.^25^ Enzymes (29.4 g/g-glucan) are added in the hydrolysis reactor to facilitate the conversion of cellulose and hemicellulose into hexose and pentose sugars, primarily glucose and xylose, respectively (Table 1 and Table S3).^25^

The lignin and remaining solid fractions of the biomass are separated after hydrolysis using a vacuum belt filter. This filtration process involves filtration, cake washing, and drying steps, with the washed water being mixed with the filtrate materials. The cake washing step minimizes losses of the IL and sugars within the solid cake. Separating the solid materials before bioconversion reduces the energy required for the bioconversion process and the size of the bioconversion reactor.

Furthermore, the IL is recovered before bioconversion using a pervaporation system^26^ because the IL used in this study can be metabolized by some of the host microorganisms. Early IL recovery simplifies downstream product recovery and purification processes. Subsequently, the solid materials are directed to the onsite energy generation unit in the case of *E. coli* and *S. cerevisiae*, whereas they are sent to the lignin depolymerization unit when using other microbial hosts, including *R. toruloides*, *P. putida*, and *C. glutamicum*, as these microorganisms can consume lignin monomers alongside sugars. The liquid fraction, which primarily contains sugars and water, is sent to the bioconversion unit.

### 2.5 Lignin Depolymerization

To capture the impacts of lignin valorization, we quantify the impact of utilizing lignin-derived aromatics as a carbon source on the minimum selling price of indigoidine and associated GHG emissions. *R. toruloides*, *P. putida*, and *C. glutamicum* are capable of utilizing aromatics. In these cases, lignin is directed to the lignin depolymerization stage rather than going directly to onsite energy generation. A mild NaOH-based treatment is employed to depolymerize lignin into its monomers, primarily *p*-coumaric, ferulic, and vanillic acids.^27^ The operating conditions, including NaOH loading of 2 wt %, a temperature of 120 °C, and a residence time of 30 min, as well as conversion rates for the lignin depolymerization, are consistent with recent work^27^ and summarized in Table 1 and Table S3. The lignin monomers generated after pretreatment are separated using a vacuum belt filter. The filtrate is then routed to the bioconversion unit, where host microorganisms, including *R. toruloides*, *P. putida*, and *C. glutamicum*, metabolize the lignin monomers along with other carbon sources, such as glucose and xylose. The remaining solid fraction is delivered to the onsite energy generation unit.

### 2.6 Bioconversion

Bioconversion of the hydrolyzed materials requires oxygen and nitrogen sources. Experiments were conducted in a shake flask and scaled up to a 2 L bioreactor,^1,12,14−16^ where dissolved oxygen was maintained by supplying air at 0.5 to 1 vvm and mostly adjusting the agitation speed. For modeling purposes, this study considered a bubble column bioreactor, which supplied high-pressure air (310.3 kPa or 45 psi).^27^ Air serves as the oxygen source, and an adequate supply is provided to maintain cellular redox balance. Corn steep liquor and diammonium phosphate are used as nutrient sources during the seed reaction for microbial growth.^24,27^ Additionally, 15 mL/L of soy sauce is added to support the growth of *C. glutamicum* that was used in the study we referenced,^1^ aiming to improve the intracellular supply of glutamine (an indigoidine precursor) and enhance the titer. It is important to note that there is no evidence that *C. glutamicum* benefits disproportionately from the addition of soy sauce relative to other organisms.

Ammonium sulfate is introduced into the primary bioconversion reactor to supply the necessary nitrogen for the host organism. It is important to note that experimental studies^1,12,14−16^ reported specific nitrogen sources in the media used. All selected microbes performed well with ammonium sulfate, except *R. toruloides*. Indigoidine production in *R. toruloides* was reported to be about twofold higher with urea compared to ammonium sulfate in the media tested. For simplicity, the process and metabolic models developed in this study considered ammonium sulfate as the nitrogen source, and the corresponding indigoidine yield. However, indigoidine yield in *R. toruloides* can be further increased with urea or other low-cost nitrogen sources that maintain a carbon–nitrogen (C/N) ratio of 8. In the case of *C. glutamicum*, calcium hydroxide is supplied to the bioreactor to control pH by converting lactic acid into calcium lactate and water, which also aids in lactic acid recovery.^28^ Following bioconversion, the entire slurry is directed to the recovery stage.

### 2.7 Indigoidine Recovery

In the recovery stage, the solid and liquid fractions of the slurry obtained from bioconversion are separated through vacuum filtration. Dimethyl sulfoxide (DMSO) and dimethylformamide (DMF) are commonly used solvents for extracting indigoidine in experimental works.^12,15,16^ A prior study^15^ also reported that tetrahydrofuran (THF) can be used as an alternative solvent for indigoidine extraction. THF was used as the extraction solvent in this study due to its easier recovery compared to DMSO and DMF. The indigoidine extraction efficiency in THF is not fully understood, requiring further investigation. We have conducted sensitivity analysis considering a wide range of extraction efficiencies (70–99%). The solid fraction is transferred to the cell lysis process, where THF is introduced.^15^ The filtrate is directed to the wastewater treatment stage, with the exception of *S. cerevisiae*. In the case of *S. cerevisiae*, the filtrate is channeled to the ethanol recovery unit, which is discussed in the coproduct recovery section (Section S3).

After the cell lysis, the cell mass, obtained by vacuum filtration, is typically routed to the onsite energy generation unit, except in the case of *C. glutamicum*. For *C. glutamicum*, the solid fraction, which also contains calcium lactate, is directed to the lactic acid recovery unit, which is discussed in the coproduct recovery section (Section S3). During this filtration process, additional THF is used to wash the cell mass, reducing the loss of indigoidine in the solid microbial cake. The washed materials are then combined with the filtrate. The filtrate–indigoidine–solvent mixture is subjected to distillation to recover THF, followed by evaporation to remove water and any remaining solvent impurities. The waste materials are sent to the wastewater treatment stage, while the recovered indigoidine is stored onsite.

### 2.8 Wastewater Treatment, Onsite Energy, and Utilities

The downstream Wastewater Treatment, Onsite Energy, and Utility stages are modeled consistent with prior works.^24,27^ The wastewater is treated using a combination of anaerobic and aerobic processes. In the onsite energy generation unit, process steam and electricity are produced using the lignin fraction of biomass, biogas obtained from the anaerobic wastewater treatment unit, and supplemental natural gas as required consistent with previous studies.^24,27^ The utility section includes a groundwater pumping system, cooling water tower, and chilled water system. These stages are further discussed in the Sections S4 and S5.

### 2.9 Scenarios and Data Inputs

We evaluated three scenarios: current, baseline (50% of theoretical yield), and optimal (90% of theoretical yield), representing the current state of technology, intermediate improvements, and future potential. The optimal scenario also accounts for improvements in upstream biomass supply and deconstruction. Carbon source utilization for each microbe was adjusted based on experimental data and projected advancements. Indigoidine yield, along with oxygen and nitrogen sources for cell redox balancing, were calculated using a genome-scale metabolic model (Section S7). Table 1 and Table S3 summarize the input data for each scenario.

### 2.10 Quantification of Minimum Selling Price and Lifecycle Greenhouse Gas Emissions

The methods employed to determine the minimum selling price and evaluate the lifecycle GHG emissions align with established approaches in prior studies. The process model is constructed using SuperPro Designer V13, where material and energy balances for each unit operation are carried out using the software’s built-in functions. Equipment size and quantity are based on the resultant material balance data. Equipment purchasing prices are calculated by considering baseline prices, changes in equipment size, and scaling exponents. The baseline size and equipment purchasing prices are sourced from recent publications. This process model accounts for variations in input parameters and their effects on material and energy flows, as well as the resulting capital and operating costs. The capital cost is adjusted to the year 2022$ using the plant cost index.

Following the compilation of capital and operating costs obtained from the models developed in SuperPro Designer, a discounted cash flow rate of return (DCFROR) analysis is conducted in Microsoft Excel. This analysis incorporates both direct and indirect overhead cost factors, in alignment with methodologies used in previous studies. The DCFROR analysis is instrumental in determining the minimum selling price of indigoidine. It considers an internal rate of return (IRR) after taxes set at 10%, a plant lifetime of 30 years, 7920 operating hours per year (equivalent to 330 days per year and 24 h per day), and an income tax rate of 21%.^27,31^ All other economic evaluation parameters are maintained in line with prior techno-economic studies.^24,27^

The lifecycle GHG footprint is calculated using Bio-Cradle-to-Grave (BioC2G), a hybrid process-based/physical unit-based input–output model documented in earlier research.^32−34^ This model comprehensively outlines the methods and data inputs. In summary, the lifecycle assessment model calculates GHG emissions by considering the input–output matrix for all relevant direct and indirect inputs/outputs, along with GHG impact vectors sourced from other LCA databases.^35−37^ Material and energy balance data generated from the process model developed in this study are major inputs to the LCA model. The onsite electricity credit is assessed based on the displacement of an equivalent amount of grid electricity (U.S. average electricity mix). Additionally, ethanol and lactic acid offset credits applied as part of system expansion were gathered from the GREET LCA model,^35^ where ethanol is assumed to displace gasoline and lactic acid assumed to displace corn-derived lactic acid. The functional unit is 1 kg of indigoidine.

## 3 Results and Discussion

### 3.1 Minimum Selling Price of Indigoidine by Microbial Host

**Figure 2:**
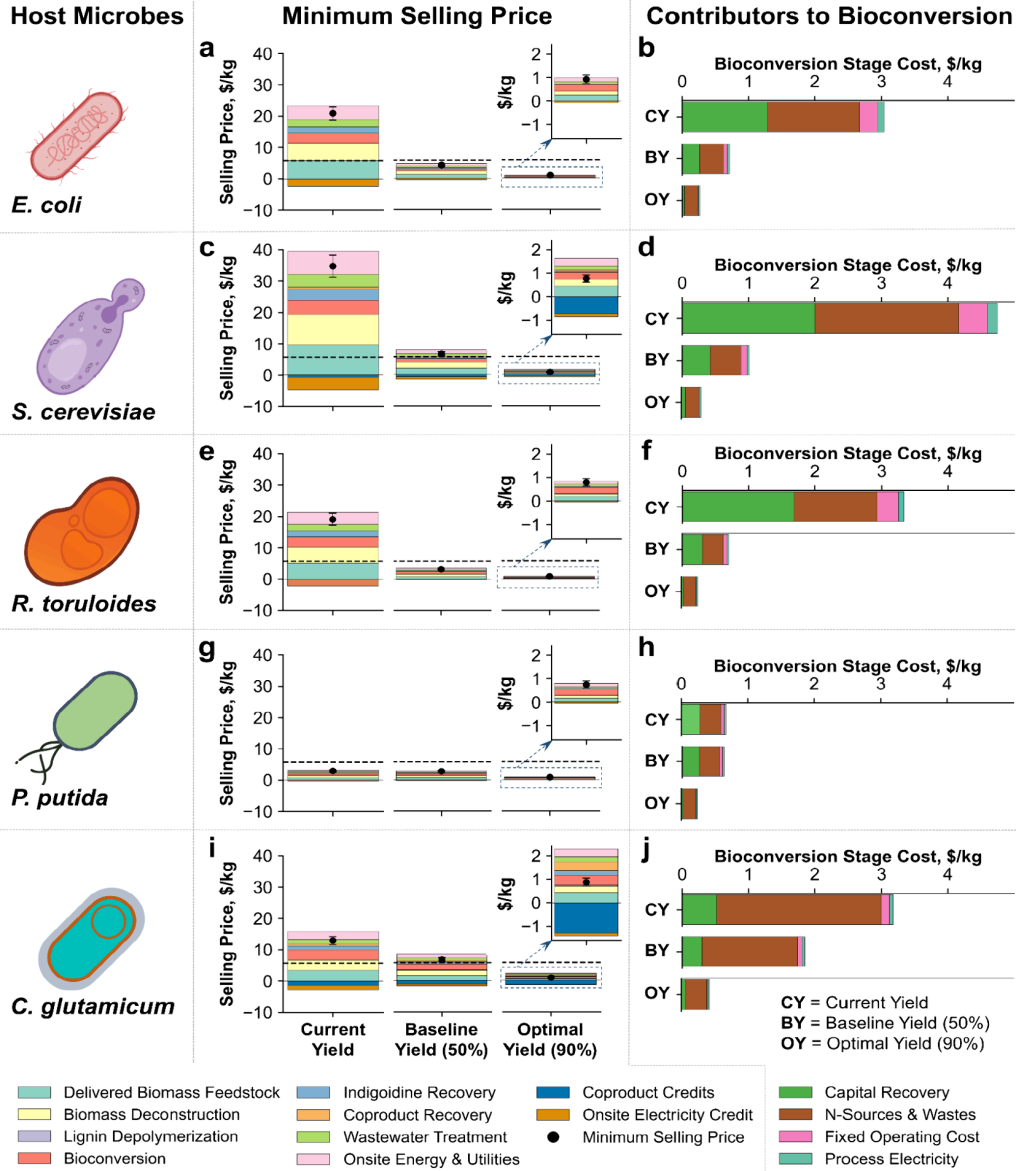
Minimum selling price (MSP) of indigoidine with different host microbes utilizing only sugars, and sugars and aromatics. In this figure, (a), (c), (e), (g), and (i) represent the MSP, while (b), (d), (f), (h), and (j) represent the contribution of the bioconversion stage to the total MSP of indigoidine produced in *E. coli*, *S. cerevisiae*, *R. toruloides*, *P. putida*, and *C. glutamicum*, respectively. The uncertainty bars illustrate the impact of a 10% variation in the data inputs presented in Table 1 and Tables S2 and S3. The horizontal dashed lines represent an average selling price of synthetic indigo dye of $5.7/kg.^8,10,11^

Figure 2 shows the minimum selling price for indigoidine across multiple hosts and scenarios. Even with currently demonstrated yields, *P. putida* can already produce indigoidine at $2.9/kg, which is below the market price of synthetic indigo of $5.7/kg (Figure 2g), assuming the two compounds are functionally equivalent per kg.

For *R. toruloides*, *E. coli*, and *S. cerevisiae*, production costs are approximately three, four, and six times higher, respectively, compared to the commercial synthetic indigo price based on the current wet lab yields. However, these microbes have the potential to produce higher amounts of indigoidine, which substantially decreases the selling price, as demonstrated in the baseline and optimal scenarios (Figure 2). Differences across host microbes at current yields can, in some cases, be more of an indication of the level of research effort devoted to them rather than an indication of their ultimate potential as commercial hosts. At 90% of theoretical maximum yield using similar cultivation parameters and media components, all hosts achieve similar minimum selling prices for indigoidine.

When further yield increases are not feasible, other process-level improvements that enhance bioproduct titer, productivity, or both become critical to reducing the minimum selling price. Increasing titer or productivity—particularly by reducing bioconversion time, improving biomass deconstruction yields at higher solid loadings, enhancing the quality of biomass feedstock (determined by carbohydrate and lignin content), or a combination of these factors—is important. These improvement opportunities are incorporated in the optimal yield scenario, resulting in a minimum selling price of indigoidine that is about five times lower than market price of synthetic indigo across all microbes.

*P. putida* is currently the top choice for producing economically viable indigoidine, because it has demonstrated the ability to utilize glucose, xylose, and aromatics with very high yield. Even the optimal indigoidine price with *P. putida* is lower than that of other microbes. However, *P. putida* does not naturally catabolize xylose and requires strain engineering, which is successfully demonstrated in a prior work.^19^ It also needs to be tested with whole plant hydrolysates. Conversely, *R. toruloides* naturally utilizes whole plant hydrolysate and achieves a similar minimum selling price to *P. putida* when the process is fully optimized, although the degree to which these organisms can achieve comparable titer, rate, and yield with ammonium sulfate remains unknown. The success of *C. glutamicum* depends on how efficiently it can produce in a minimal medium without supplemental commercial soy sauce, and how valuable the lactic acid coproduct is given market dynamics. Particularly if simple sugars or a mixture of plant-derived sugars are the feedstock of choice, *E. coli* and *S. cerevisiae* can be attractive. However, all these selected microbes need to be tested with whole plant hydrolates or mixed carbon sources. These results suggest that the choice of microbes should be made based on the available carbon sources, how efficiently and quickly pathways can be optimized for a given microbe, and the value of coproducts, where applicable.

Across the potential host microbes, a key difference in the bioconversion stage is the oxygen and nitrogen levels required for cellular redox balancing. The bioconversion stage’s cost analysis shows that capital recovery costs and nitrogen source expenses are the major contributors. One obvious strategy for improving these costs is to increase the rate of production. Reducing the residence time during bioconversion lowers capital costs (Figure S2) by reducing the required size or number of bioreactors and minimizing the energy consumption of the bioreactors. Similarly, reducing oxygen requirements is crucial for minimizing energy consumption (sparging and agitation drive the energy demand in the bioreactors). These parameters depend on the choice of microbes, and future research should focus on selecting microbes based on their ability to grow efficiently in minimal media without expensive nitrogen sources, perform effectively under lower oxygen levels, translate oxygen and nitrogen use into maximum product yield, and achieve high rates of indigoidine production. These exercises can sometimes be important to identify cheaper medium parameters and their optimal ratios.^38^

### 3.2 Lifecycle Greenhouse Gas Footprint of Indigoidine by Microbial Host

**Figure 3:**
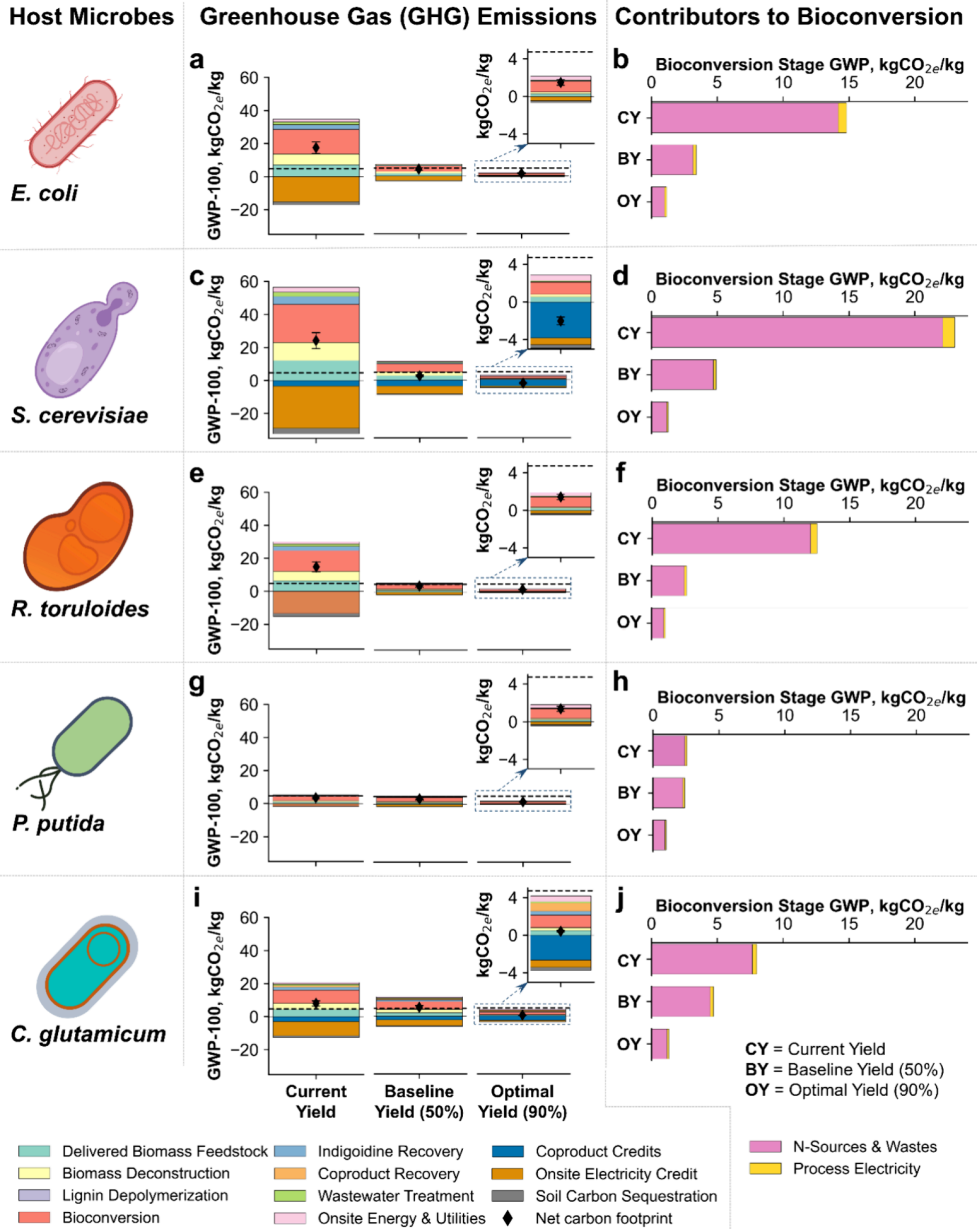
Lifecycle greenhouse gas emissions of indigoidine with different host microbes utilizing only sugars, and sugars and aromatics. In this figure, (a), (c), (e), (g), and (i) represent the GHG emissions, while (b), (d), (f), (h), and (j) represent the contribution of the bioconversion stage to the total GHG emissions of indigoidine produced in *E. coli*, *S. cerevisiae*, *R. toruloides*, *P. putida*, and *C. glutamicum*, respectively. The uncertainty bars illustrate the impact of a 10% variation in the data inputs presented in Table 1 and Tables S2 and S3. The horizontal dashed lines represent a conservative greenhouse gas emissions footprint of synthetic indigo dye of 4.72 kgCO_2e_/kg (Table S1), with estimated values ranging from 4.7 to 13.8 kg CO_2e_/kg (Section S1).

Figure 3 provides a detailed analysis of overall GHG emissions and contributors to bioconversion stage GHG emissions for five microbial hosts: *E. coli*, *S. cerevisiae*, *R. toruloides*, *P. putida*, and *C. glutamicum*. Similar to the minimum selling price of indigoidine, yield improvements lead to substantial reductions in both overall GHG emissions and bioconversion stage emissions across all microbes. *E. coli* and *S. cerevisiae* have not yet achieved yields comparable to *R. toruloides*, *P. putida*, and *C. glutamicum*, and this translates into higher lifecycle GHG emissions based on their state of technology. Once yields are optimized to 90% of theoretical, all host microbes achieve comparable GHG footprints for indigoidine.

The reduction in GHG emissions in the optimal scenario, which incorporates advances beyond just yield improvements, is attributable to other process improvements that increase the titer and productivity of indigoidine. The results indicate that a system expansion approach to coproduct accounting results in a net-negative GHG foorprint of indigoidine in the case of *S. cerevisiae*, which coproduces ethanol, and a near-zero GHG footprint in the case of indigoidine production via *C. glutamicum* (which coproduces lactic acid). However, these lower emissions are largely dependent on the carbon footprint credit of coproducts, where ethanol can displace gasoline and lactic acid can displace lactic acid produced from the fermentation of corn-derived sugars. If, for example, ethanol is assigned an offset credit corresponding to a lower-carbon product (e.g., cellulosic ethanol), the net GHG footprint of indigoidine from *S. cerevisiae* may no longer be net-negative.

Another notable result is that even an intermediate target of 50% of the theoretical yield is sufficient to achieve GHG emissions for renewable indigoidine that are lower or similar to synthetic indigo dye, particularly when coproduct credits are considered. This is especially true for microbes with high productivity, those requiring lower oxygen and nitrogen inputs, or a combination of these factors. This indicates that increasing yield, along with enhancing productivity, is crucial for reducing overall GHG emissions.

Among the selected microbes, there are substantial differences in GHG emissions in the bioconversion stage, particularly due to use of different levels of nitrogen sources. Therefore, exploring low-carbon nitrogen sources is important to reduce both stage-specific and overall GHG emissions. Additionally, supplying only the oxygen sufficient for cellular redox balancing and reducing bioconversion time can lower the GHG emissions associated with electricity usage.

### 3.3 Value of Suppressing Coproduct Production Pathways

**Figure 4:**
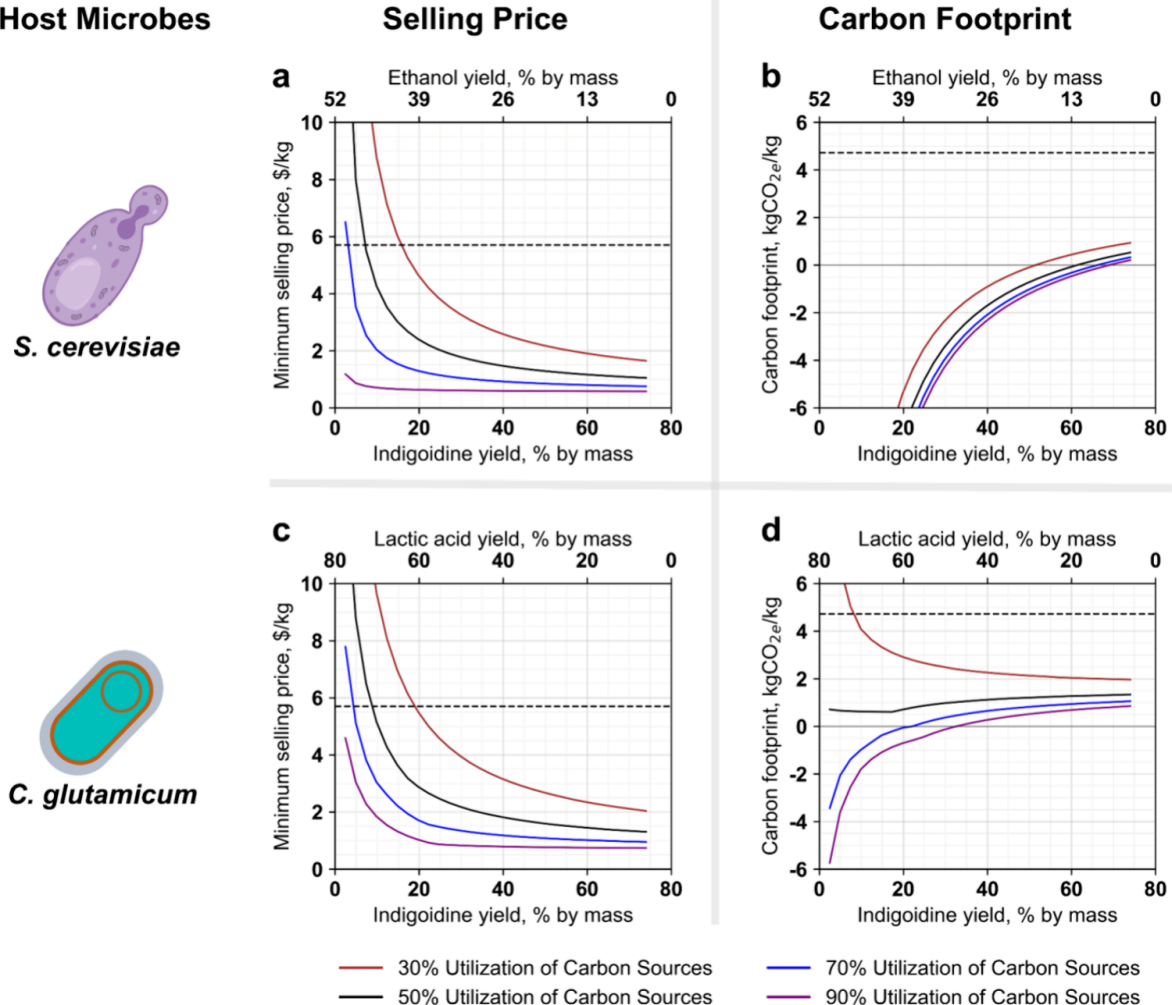
Production cost and greenhouse gas emissions of indigoidine as a function of product and coproduct yields (mass % yield per mass of carbon sources). The horizontal dashed and dotted lines represent an average selling price of $5.7/kg^8,10,11^ (a, c) and greenhouse gas emissions (Table S1) of 4.72 kgCO_2e_/kg (b, d) of synthetic indigo dye, respectively. The lifecycle GHG results reflect system expansion for coproduct accounting, using indigoidine as the primary product.

In the examples used, *S. cerevisiae* and *C. glutamicum* generated ethanol and lactic acid, respectively, along with indigoidine, and both of these side products are expected to sell for comparatively lower prices. The combination of product and coproduct fractions affects the overall cost and GHG emissions of indigoidine. We conducted this analysis for a range of carbon source conversion efficiencies, ranging from 30 to 90%, while keeping other process parameters aligned with the optimal yield scenario values. We considered potential scenarios where carbon sources are fully diverted to indigoidine production, achieving maximum indigoidine yield with minimal or nearly zero coproduct formation, and vice versa (Figure 4). The remaining carbon in these scenarios goes to cell mass and CO_2_. The carbon sources include lignocellulosic sugars (pentose and hexose) for *S. cerevisiae* and lignocellulosic sugars and lignin-derived aromatics for *C. glutamicum*; however, lignin monomers are solely diverted to indigoidine production as model lignin monomer (*para*-coumarate) considered in this work does not produce lactic acid coproduct. Nitrogen input is adjusted for each scenario based on the fraction of carbon routed to indigoidine. The results highlight the substantial impacts of carbon source utilization and yield improvements on both the MSP and carbon footprint for these microbial hosts.

For both microbes, at low-carbon source utilization (30%), the minimum selling price of indigoidine gradually decreases as indigoidine yield increases and coproduct yield decreases. As the efficiency of carbon source utilization increases, the cost curve drops sharply, revealing threshold points. For *S. cerevisiae*, at 90% carbon source utilization efficiency, the results show that achieving just a 10% indigoidine yield by mass is sufficient to reach a lower selling price, beyond which the selling price does not change substantially. Under these conditions, an indigoidine biorefinery could divert a large fraction of sugars into ethanol, resulting in approximately 45% ethanol by mass. For *C. glutamicum* with 90% carbon source utilization efficiency, the indigoidine yield threshold is 20% by mass (combining both sugars and aromatics), with a corresponding lactic acid yield of 60% by mass (from glucose and xylose). Future strain engineering is required to achieve these combinations of indigoidine and coproducts. If *S. cerevisiae* and *C. glutamicum* are engineered accordingly, indigoidine could become a valuable coproduct in ethanol and lactic acid biorefineries. Such scenarios are possible for the other microbial hosts also, which can be also engineered for coproducts.

In contrast to production cost, the carbon footprint of indigoidine produced in *S. cerevisiae* increases as indigoidine yield rises (with corresponding decreases in ethanol production). This is primarily due to the fact that ethanol offsets gasoline in our analysis. The results show a negative carbon footprint for indigoidine until the yield reaches 48% by mass at a 30% sugar utilization rate. This threshold increases with higher sugar utilization, reaching 67% by mass when 90% of sugars are utilized. At the lower-cost threshold of 10% by mass, the carbon footprint of indigoidine is below −6 kg CO_2e_/kg, indicating that lower indigoidine production maximizes carbon reduction benefits.

The GHG footprint of indigoidine produced in *C. glutamicum* exhibits a more complex relationship with indigoidine yield. When carbon source conversion efficiency to products is low (30%), increasing the diversion of sugars to lactic acid at the expense of indigoidine production increases the GHG footprint of indigoidine. When carbon source utilization efficiency is high (90%), diverting more sugars to lactic acid at the expense of indigoidine yield decreases the GHG footprint of indigoidine (because the coproduct offset credit for lactic acid is sufficiently large to produce a net-negative GHG footprint). However, this result is a function of the coproduct accounting method; one may argue that applying an offset credit for lactic acid when it comprises the majority of the mass output is less defensible than other methods such as energy, mass, or market value-based allocation. This result highlights the challenges of applying coproduct allocation consistently for biological processes with multiple products. The GHG footprint stabilizes when the carbon source consumption reaches around 50% of its initial amount. When most of the carbon sources are utilized (70 and 90% of its initial amount), a negative carbon footprint is achieved at lower indigoidine yields. However, the carbon footprint increases as indigoidine yield continues to rise. For 90% sugar utilization, the carbon footprint of indigoidine could turn positive when the yield exceeds 27% by mass. At the lower-cost threshold (with an indigoidine yield of 20% by mass), carbon-negative indigoidine (−0.5 kg CO_2e_/kg) can be produced in *C. glutamicum*.

### 3.4 Improving the Economic and Environmental Value of Microbially Produced Indigoidine

The findings emphasize that while enhancing microbial host performance is essential, achieving higher titers, rates, and yields requires system-level improvements. This section is elaborated further in Section S9. The following paragraphs briefly summarize the main points.

Tailoring plant feedstock engineering to optimize biomass composition to match catabolic capabilities of the microbial conversion platform is crucial for aligning with downstream conversion pathways and biorefinery configurations. Lignin, when not fully utilized, can serve as an energy source, but burning it in its moist form is inefficient, potentially raising capital costs. Fine-tuning biomass composition can lower these costs and improve energy efficiency by increasing the carbohydrate fraction, boosting sugar concentrations entering bioreactors, and enhancing productivity without additional biomass deconstruction improvements (Figures S3 and S4).

Increasing solid loading during biomass deconstruction can further elevate sugar concentrations, addressing the common challenge of low sugar yields in lignocellulosic biorefineries. This approach avoids additional costs related to sugar concentration units and enhances economic benefits while reducing GHG emissions (Figures S3 and S4). However, microbial bioconversion efficiency at high sugar concentrations remains a challenge, specifically in aerobic cultivations. Identifying microbes that can efficiently utilize concentrated carbon sources is crucial for maintaining yield and productivity. Additionally, exploring cost- and energy-efficient methods for indigoidine extraction is important, as it has the potential to substantially impact both cost and GHG emissions (Figures S3 and S4).

This study highlights the necessity of focusing on high-yield, high-value products, like indigoidine, to ensure economic viability. Selecting microbes capable of efficiently utilizing available carbon sources, including lignin-derived aromatics, is essential, but it requires careful consideration of energy and material demands. It is also possible to reduce costs and emissions by moving away from solely maximizing single products and instead taking advantage of efficiently separable coproduct pathways.

## 4 Conclusions

This study highlights the critical role of microbial host selection on the economic and environmental performance of indigoidine production. Matching feedstock composition with the microbial catabolic profile is essential to maximize both economic and environmental benefits. Microbes that convert a larger fraction of carbon sources (sugars and aromatics) into indigoidine, such as *P. putida*, result in lower production costs, provided that lignin can be cost-effectively deconstructed into bioavailable intermediates that are not toxic to the host. Conversely, microbes that produce coproducts along with indigoidine, such as *S. cerevisiae*, can achieve lower or even carbon-negative indigoidine production, depending on how coproducts are accounted for. This highlights that an easily separable, high-value coproduct could be crucial for reducing both the production cost and GHG emissions of the product. If reducing GHG emissions is a priority, lignin can be combusted onsite, and the selection of microbial hosts capable of coproducing and displacing carbon-intensive petroleum products becomes critical. In this study/using conversion data available, *P. putida* is identified as a leading choice for cost-effective production due to its high yield and efficient utilization of glucose and aromatics. However, *R. toruloides* and *C. glutamicum* also present competitive alternatives depending on specific process requirements and coproduct values. The results emphasize the need for integrating microbial engineering, optimized biomass composition, and process enhancements to achieve both economic viability and environmental sustainability in indigoidine production. It is essential to explore various product and coproduct combinations, acknowledging that some microbes naturally produce specific products while others require engineering. For intracellular products like indigoidine, yield is a key factor, as it substantially influences downstream extraction and recovery costs. Additionally, for rate-limiting host microbes, switching from a conventional stirred-tank bioreactor to a bubble column bioreactor is important, although reducing oxygen requirements remains crucial to lowering air supply cost and energy. Further research should focus on pathways that combine high-energy-density biofuel production with high-value biochemicals or biomaterials, supported by integrated TEA and lifecycle assessment.

## Supporting Information Available

GHG emissions calculations for synthetic indigo, additional methodological details, detailed data inputs, CAPEX and OPEX results, sensitivity analysis results, and detailed process equipment sizes and costs (PDF)
