# Supplementary material for: Microbial Pathways for Cost-Effective Low-Carbon Renewable Indigoidine
Source: ACS Sustain Chem Eng. 2025 Feb 14;13(8):3300–10. doi: 10.1021/acssuschemeng.4c09962 (PMC11881132; doi:10.1021/acssuschemeng.4c09962)
Supplement: Supplementary file 1 — sc4c09962_si_001.pdf [file sc4c09962_si_001.pdf]

Supporting Information

Microbial Pathways for Cost-Effective Low-Carbon Renewable Indigoidine

Nawa Raj Baral,[a,b,*] Deepanwita Banerjee,[a,b] Thomas Eng,[a,b] Blake A. Simmons,[a,b] Aindrila Mukhopadhyay,[a,b] and Corinne D. Scown[a,b,c,d]

[a]Joint BioEnergy Institute, Lawrence Berkeley National Laboratory, Berkeley, California 94720, United States.
[b]Biological Systems and Engineering Division, Lawrence Berkeley National Laboratory, Berkeley, California 94720, United States.
[c]Energy Analysis and Environmental Impacts Division, Lawrence Berkeley National Laboratory, Berkeley, California 94720, United States.
[d]Energy & Biosciences Institute, University of California, Berkeley, California 94720, United States
*Corresponding author; nrbaral@lbl.gov

Supporting Information includes:
Number of pages: 21
Number of figures: 4
Number of tables: 4

S1. Calculation of Greenhouse Gas Emissions Footprint of Synthetic Indigo Dye

Considering the global market for synthetic indigo dye, which is approximately 80 to 110 thousand metric tons per year.[1,2] The carbon footprint of synthetic indigo is estimated at 4.72 $kgCO_{2e}/kg$, assuming a dye usage rate of 20 g/kg of fiber (Table S1). If dye consumption decreases to 10 g/kg of fiber,[3] the carbon footprint increases to 9.4 $kgCO_{2e}/kg$ (Table S1). Based on the material and energy inputs reported for synthetic indigo production in a previous study, [4] along with GHG emissions data from the GREET LCA model, [5] we estimate the carbon footprint to be 13.8 $kgCO_{2e}/kg$. Another study [6] reported GHG emissions for synthetic indigo dye at 10.8 kg CO_{2e}/kg. Given this range of carbon footprints and the annual production of synthetic indigo dye, we estimate the corresponding annual GHG emissions to be between 0.37 and 1.52 million metric tons of CO_2.

Table S1. Carbon footprint of synthetic indigo dye

Description	Unit	Value
GHG emissions for yarn production, dyeing, weaving and knitting[7]	$kgCO_{2e}$/kg-fiber	9.60
Contribution from dyeing and finishing[8]	%	47.20
GHG emissions for dyeing and finishing$^{\varphi}$	$kgCO_{2e}$/kg-fiber	4.53
Contribution from dyeing process[8]	%	41.67
GHG emissions for dyeing process$^{\varphi}$	$kgCO_{2e}$/kg-fiber	1.89
Dye contribution to the total GHG emissions from dyeing process[9]	%	5.00
GHG emissions from dye$^{\varphi}$	$kgCO_{2e}$/kg-fiber	0.09
Dye consumption[7]	g/kg-fiber	20.00
GHG emissions from synthetic dye$^{\varphi}$	$kgCO_{2e}$/kg-dye	4.72

$^{\varphi}$Calculated value

S2. Biomass Sorghum Production and Supply

The biomass sorghum production and supply unit encompasses biomass production, harvesting, transportation, and storage, which has been adapted from our previous work.[10] The harvesting process involves a series of field operations, including windrowing, field drying, conditioning (which involves breaking stems to expedite dry-down and reduce energy consumption during baling), baling, and stacking biomass bales at the field's edge. The trucking distance from the field to the biorefinery was determined based on a typical biomass sorghum yield of 17.9 bone-dry metric tons per hectare (~8 bone-dry tons per acre),[10,11] a biomass sorghum production area around the biorefinery of 5%,[10] and a dry material loss of 11.6%[10] across the entire supply chain. The moisture content of the biomass bales delivered at the biorefinery gate is assumed to be 20%.[10] All the necessary data inputs used to calculate the cost of biomass sorghum production and supply are detailed in a previous study.[10]

This biomass production and supply model was developed in a manner consistent with other biomass feedstock supply models, including corn stover, miscanthus, and switchgrass.[12,13] Unlike corn stover, where production costs and associated greenhouse gas (GHG) emissions can be entirely attributed to corn grain, for non-grain biomass sorghum considered in this work, production costs and associated GHG emissions were solely allocated to biomass. This

allocation encompasses expenses such as land rent, fuel costs, labor costs, fertilizer costs, herbicide costs, and other establishment costs, including seed and machinery costs.

In addition to biomass production, our cost model for other logistical operations, such as harvesting, transportation, and storage, is based on a standard cash flow analysis.[10] This analysis incorporates capital investments, ownership costs (including depreciation, interest, taxes, insurance, and housing), and operating costs (comprising repair and maintenance, fuel, lubrication, labor, and consumable materials like string, tarp, and plastic wrap). The materials and fuels obtained from the cost model were used to calculate GHG emissions associated with biomass production and supply.

S3. Coproduct Recovery

Among the selected microbial hosts, *S. cerevisiae* produces ethanol as a co-product, while *C. glutamicum* produces lactic acid as a co-product. Ethanol is recovered using a process that involves distillation followed by molecular sieve adsorption.[14] The distillation separates ethanol into a near-azeotropic mixture with water, which is subsequently purified to a concentration of 99.5% using vapor-phase molecular sieve adsorption.[14] Solids recovered from the distillation bottoms are directed to the onsite energy generation unit, while the liquid portion is sent to wastewater treatment.

Although solvent and membrane-based lactic acid recovery processes have been investigated in recent years,[15] we consider the proven $Ca(OH)_2$-based precipitation method to recover lactic acid, consistent with the methods discussed in prior reviews [15–17] and a TEA study [18]. Briefly, the solid fraction obtained after indigoidine recovery primarily consists of cell mass and calcium lactate. This fraction undergoes a series of processes to recover lactic acid. The solid mixture is first sent to the acidification reactor, where calcium lactate is converted back into its acidic form using a concentrated sulfuric acid solution. After acidification, the solid fraction is separated using a vacuum belt filter and then routed to the onsite energy generation unit.

The filtrate solution, which contains lactic acid and impurities, is cooled down and then sent through an ion exchange process, followed by an activated carbon column for purification. All waste streams from the ion exchangers and the activated carbon columns are collected, neutralized, and sent to the wastewater treatment unit.

The recovered lactic acid is further concentrated using a multi-effect evaporator and then purified using two subsequent distillation columns.[15,18] The bottom of the first column, which contains lactic acid and some impurities, is fed into a second distillation column. The distillate from the first column is combined with the condensate from the evaporator, which contains a small amount of lactic acid and is recycled back into the process.

The lactic acid product is collected in the distillate from the second column, cooled down to room temperature and stored onsite. The bottom of the second column, which contains impurities and small amounts of lactic acid, is sent to the wastewater treatment unit.

S4. Wastewater Treatment

The process model for wastewater treatment aligns with our previous study [19] and with similar prior studies[14,20] conducted by the National Renewable Energy Laboratory (NREL). The wastewater is treated using a combination of anaerobic and aerobic processes. Biogas generated during the anaerobic wastewater treatment stage is directed to onsite energy generation, while the reclaimed process water is utilized in upstream processes. Solid waste is transported to a landfill site for disposal, and the model assigns a waste disposal cost of $42.13 per metric ton.[14]

S5. Onsite Energy and Utility

In the onsite energy generation unit, process steam and electricity are produced using the lignin fraction of biomass and biogas obtained from the anaerobic wastewater treatment unit, consistent with previous studies.[14,20] When lignin is unavailable or insufficient to meet the facility's heat and power demands, natural gas is supplied as a supplementary heat source. Any excess steam generated beyond the facility's heating requirements is directed to the turbo-generator to produce electricity. The solid waste generated during the onsite energy generation stage is managed similarly to the solid waste from the wastewater treatment stage.[14]

In addition to process steam and electricity, the biorefinery requires cooling water, chilled water, and process water. The utility section includes a groundwater pumping system, cooling water tower, and chilled water system. The assumptions and system layout for this stage are in line with the previous studies.[14,20]

S6. Biorefinery Size

Figure S1 represents a typical economic size of indigodine biorefinery considering indigoidine production in P. putida at experimentally reported indigoidine yield. This scale is consistent with a typical nth plant analysis. However, given the comparatively smaller size of the indigo market relative to commodity chemicals and fuels, it is possible that smaller scales may be viable and this warrants further analysis. Additionally, further testing of indigoidine's properties as a replacement for synthetic indigo dye can ensure that the volume of indigoidine needed to replace a functionally equivalent volume of synthetic indigo is adjusted as needed.

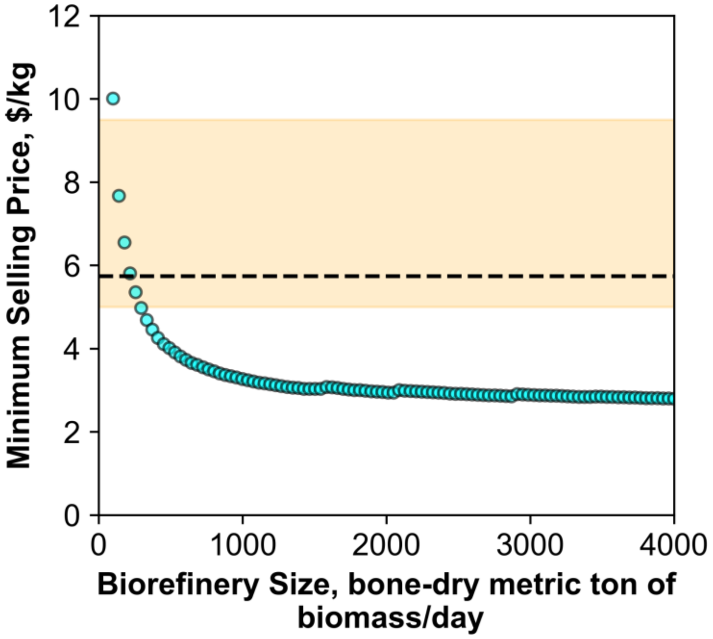

Figure S1. Minimum selling price of indigoidine as a function of biorefinery size. The dashed line represents the market price of synthetic indigo dye, and the shaded area indicates its variation primarily due to quality.

S7. Calculation of Biological Theoretical Yield

We calculated indigoidine yield, as well as oxygen and nitrogen requirements, using recent genome-scale metabolic models for *Escherichia Coli* (iML1515), *Saccharomyces Cerevisiae* (Yeast 8), *Rhodosporidium Toruloides* (iRhto1108C), *Pseudomonas Putida* (iJN1463), and *Corenybacterium Glutamicum* (iCGB21FR), along with Flux Balance Analysis (FBA) [21] based on reaction stoichiometry, redox balance and ATP maintenance. FBA simulations were performed using the COBRA Toolbox v.3.0 [22] in MATLAB R2017b, with the GLPK (https://gnu.org/software/glpk) or Gurobi Optimizer 8.1 (http://www.gurobi.com/) as the linear optimization solver. The set of reactions accounting for the heterologous production of indigoidine was added to each of the models using the *addindigoidine.m* script (Supplementary file). The uptake rate for each of the carbon sources was fixed at X mmol/gDCW/hr, based on the minimal media boundary conditions/constraints. The resulting indigoidine yields are summarized in Table S2.

Table S2. Yields of indigoidine, succinate, lactate, ethanol, and other byproducts in different microbes

Carbon sources	Microbes	Unit	Indigoidine	CO_2	O_2	Succinate	L-Lactate	Ethanol
Glucose	*E. coli*	wt%	0.55	0.49	0.60	-	-	-
	S. cerevisiae	wt%	0.34	0.37	0.29	-	-	0.26
	R. toruloides	wt%	0.68	0.03	0.50	-	-	-
	P. putida	wt%	0.70	0.21	0.56	-	-	-
	C. glutamicum	wt%	0.28	0.04	0.18	0.15	0.48	-
Xylose	*E. coli*	wt%	0.55	0.42	0.51	-	-	-
	S. cerevisiae	wt%	0.25	0.37	0.21	-	-	0.26
	R. toruloides	wt%	0.68	0.23	0.42	-	-	-
	P. putida	wt%	0.74	0.12	0.45	-	-	-
	C. glutamicum	wt%	0.26	0.11	0.18	-	0.45	-
Aromatics	*E. coli*	wt%	-	-	-	-	-	-
	S. cerevisiae	wt%	-	-	-	-	-	-
	R. toruloides	wt%	0.90	4.69	8.60	-	-	-
	P. putida	wt%	0.99	3.77	8.99	-	-	-
	C. glutamicum	wt%	0.79	5.77	9.30	-	-	-

Table S3. Major inputs used to develop the field-to-indigoidine process model in this study

Parameter	Unit	State-of-technology	Near-term	Optimal future case	Range & Std. Dev.	Prob. Dist.
Biorefinery size[α]	bdt/day	2000	2000	2000	(2000, 2000)	None
Feedstock cost[10]	$/bdt	118.17	118.17	87.48	(65, 147.71)	Triangular
GHG emission[10]	$kgCO_{2e}$/bdt	144.75	144.75	95.64	(63.62, 180.93)	Triangular
Soil organic carbon sequestration[23]	$kgCO_{2e}$/bdt	-46.00	-46.00	-77.69	(-192.64, -46)	Triangular
Moisture[10]	%	20.00	20.00	20.00	(20, 30)	None
**Biomass Composition[24,25]**						
Acetate	wt%	2.20	2.20	0.90	(0.9, 2.9)	None
Ash	wt%	4.00	4.00	2.20	(2.2, 10.94) & 3.49	None
Cellulose	wt%	35.40	35.40	40.00	(20.5, 44.02) & 5.31	Lognormal
Hemicellulose	wt%	20.70	20.70	29.79	(14.5, 30) & 3.8	Lognormal
Lignin	wt%	21.00	21.00	9.89	(8, 24) & 2.7	Lognormal
Protein	wt%	4.39	4.39	5.16	(3.88, 6.19) & 0.68	None
**Biomass Deconstruction[24]**						
Solid loading for pretreatment	wt%	30.00	30.00	40.00	(20, 40)	Uniform
Ionic liquid (IL) loading	wt%	5.00	5.00	5.00	(2.5, 10)	Uniform
IL cost	$/kg	2.00	2.00	1.00	(0.5, 5)	Triangular
Pretreatment time	h	3.00	3.00	1.00	(1, 3.4)	Uniform
Pretreatment temperature	°C	140.00	140.00	140.00	(140, 140)	None
IL recovery	wt%	95.00	95.00	98.00	(90, 99)	None
Acetate to acetic acid	wt%	95.00	95.00	100.00	(90, 100)	Uniform
Sulfuric acid loading	kg/kg-IL	0.15	0.15	0.10	(0.07, 0.2)	Uniform
Sulfuric acid cost	$/kg	0.14	0.14	0.14	(0.14, 0.28)	None
Enzyme loading	mg/g-glucan	29.41	29.41	10.00	(7, 60)	Triangular
Initial solid loading for hydrolysis	wt%	20.00	20.00	25.00	(10, 30)	Uniform
Cellulose to glucose	wt%	75.86	75.86	95.00	(71.41, 99)	Uniform
Xylan to xylose	wt%	60.76	60.76	90.00	(58.7, 99)	Uniform
Hydrolysis time	h	72.00	72.00	48.00	(48, 84)	Uniform
Enzyme price	$/kg-protein	5.00	5.00	5.00	(3.5, 6)	Triangular
Hydrolysis temperature	°C	50.00	50.00	50.00	(50, 50)	None
**Lignin Depolymerization**						
NaOH loading rate[20]	%	2.00	2.00	2.00	(1.6, 5)	Uniform
Temperature[20]	°C	120	120	120	(120, 120)	None
Residence time[20]	h	0.5	0.5	0.5	(0.5, 1)	Uniform
NaOH cost[20]	$/kg	0.53	0.53	0.53	(0.5, 0.6)	Uniform
Lignin to lignin monomer[20,ψ]	%	26.70	26.70	50.00	(10, 90)	Uniform
**Bioconversion [14,20,25]**						
Inoculum cost	$/kg	0.0037	0.0037	0.001	(0.001, 0.1)	Triangular
Inoculum loading	%	10.00	10.00	5.00	(4, 10)	Triangular
Corn steep liquor (CSL) cost	$/kg	0.07	0.07	0.07	(0.06, 0.08)	Triangular
Diammonium phosphate (DAP) cost	$/kg	0.36	0.36	0.36	(0.29, 1.1)	Triangular
CSL loading	wt%	0.25	0.25	0.25	(0.16, 0.3)	Uniform
DAP loading	g/L	0.33	0.33	0.33	(0.24, 0.35)	Uniform
Ammonium sulfate cost	$/kg	0.18	0.18	0.17	(0.13, 0.22)	None
Indigoidine production in E. Coli[26,β]						
Ammonium sulfate loading	g/L	26.20	34.2	102.50	(26.2, 102.5)	None
Air supply	m^3/sec	9.20	16.98	42.50	(9.2, 42.5)	None
Air pressure	kPa	310.26	310.26	310.26	(310.26, 310.26)	None
Bioconversion time	h	72.00	72.00	48.00	(36, 84)	Triangular
Glucose-to-indigoidine	wt%	8.26	27.55	49.60	(2.7, 52.4)	Uniform
Xylose-to-indigoidine	wt%	0.00	27.27	49.10	(0, 51.8)	Uniform

Contd.

Table S3. Contd.

Parameter	Unit	State-of-technology	Near-term	Optimal future case	Range & Std. Dev.	Prob. Dist.
Indigoidine production in S. cerevisiae[27,β]						
Ammonium sulfate loading	g/L	26.70	27.01	64.75	(26.7, 64.75)	None
Air supply	m^3/sec	8.00	8.86	16.46	(8, 16.46)	None
Air pressure	kPa	310.26	310.26	310.26	(310.26, 310.26)	None
Bioconversion time	h	72.00	72.00	48.00	(36, 84)	Triangular
Glucose-to-indigoidine	wt%	4.90	17.22	30.99	(1, 32.72)	Uniform
Xylose-to-indigoidine	wt%	0.00	12.40	22.32	(0, 23.56)	Uniform
Indigoidine production in R. toruloides[28,β]						
Ammonium sulfate loading	g/L	32.45	42.5	133.88	(32.45, 133.88)	None
Air supply	m^3/sec	9.80	19.05	39.46	(9.8, 39.46)	None
Air pressure	kPa	310.26	310.26	310.26	(310.26, 310.26)	None
Bioconversion time	h	120.00	120.00	48.00	(36, 144)	Triangular
Glucose-to-indigoidine	wt%	9.31	33.75	60.76	(6.7, 64.13)	Uniform
Xylose-to-indigoidine	wt%	0.00	33.89	61.00	(0, 64.39)	Uniform
Lignin monomers-to-indigoidine	wt%	0.00	44.88	80.78	(0, 85.27)	Uniform
Indigoidine production in P. putida[29,β]						
Ammonium sulfate loading	g/L	37.68	46.8	142.60	(37.68, 142.6)	None
Air supply	m^3/sec	11.60	19.03	35.89	(11.6, 35.89)	None
Air pressure	kPa	310.26	310.26	310.26	(310.26, 310.26)	None
Bioconversion time	h	116.00	116.00	48.00	(36, 144)	Triangular
Glucose-to-indigoidine	wt%	33.00	35.13	63.24	(14, 66.75)	Uniform
Xylose-to-indigoidine	wt%	32.00	37.20	66.96	(0, 70.67)	Uniform
Lignin monomers-to-indigoidine	wt%	73.91	49.44	88.99	(0, 93.94)	Uniform
Indigoidine production in C. glutamicum[30,β]						
Ammonium sulfate loading	g/L	26.27	26.34	62.20	(26.27, 62.2)	None
Soy sauce loading	mL/L	15.00	15.00	15.00	(15, 15)	Triangular
Soy sauce price[ϑ]	$/kg	7.70	7.70	7.70	(1.5, 12)	Triangular
Air supply	m^3/sec	8.17	12.97	19.60	(8.17, 19.6)	None
Air pressure	kPa	310.26	310.26	310.26	(310.26, 310.26)	None
Bioconversion time	h	51.00	51.00	48.00	(36, 84)	Triangular
Glucose-to-indigoidine	wt%	14.00	13.78	24.79	(2.7, 26.17)	Uniform
Xylose-to-indigoidine	wt%	0.00	13.22	23.81	(0, 25.13)	Uniform
Lignin monomers-to-indigoidine	wt%	0.00	39.55	71.19	(0, 75.15)	Uniform
**Product & Coproduct Recovery**						
Indigoidine recovery[δ]	%	95.00	95.00	98.00	(90, 99)	Triangular
Tetrahydrofuran loading[γ]	%	50.00	50.00	40.00	(40, 50)	Triangular
Tetrahydrofuran loss[δ]	%	2.00	2.00	1.00	(1, 3)	Triangular
Tetrahydrofuran cost[ϑ]	$/kg	3.31	3.31	2.00	(3.31, 4)	Triangular
Lactic acid recovery[δ]	%	95.00	95.00	98.00	(90, 99)	Triangular
Ethanol recovery[δ]	%	95.00	95.00	98.00	(90, 99)	Triangular
Calcium hydroxide cost[20]	$/kg	0.20	0.20	0.20	(0.1, 0.4)	Triangular
**Wastewater Treatment** [14]						
Organic matter to biogas	%	86.00	86.00	86.00	(86, 91)	Uniform
Nutrient loading for AD	wt%	0.05	0.05	0.02	(0.01, 0.06)	Uniform
Nutrient cost	$/kg	0.70	0.70	0.45	(0.3, 0.7)	Triangular
**Onsite Energy and Utilities**[14]						
Boiler chemicals cost	$/kg	5.00	5.00	5.00	(4, 6)	Triangular
Natural gas cost	$/kg	0.22	0.22	0.22	(0.22, 0.44) & 0.1	Normal
Water cost	$/kg	0.00022	0.00022	0.00022	(0.0001, 0.0004)	Triangular
Clean-in-place chemicals cost	$/kg	0.53	0.53	0.35	(0.35, 0.65)	Triangular

bdt = bone-dry metric ton per day. [α]Assumed for analysis in this work (Fig. S1). [Ψ]Monomer yield is calculated based on the mass balance data reported in a prior modeling study,[20] a range of experimental monomer yields summarized in a previous review,[31] and similar work[32] conducted by JBEI researchers on ionic liquid-pretreated biomass. We considered the conversion of soluble lignin after ionic liquid pretreatment (32% of the initial lignin[24]) of 53%[20] and a

range of 8-20% conversion of the remaining lignin to monomers,[20,31] yielding an average of 26.7% conversion of the initial lignin to monomers. $^{\beta}$Yields are calculated in this study using genome-scale metabolic models (Table S2). $^{\delta}$Assumed similar to recovery of other biofuels or bioproducts.[14,20] $^{\gamma}$Assumptions for the analysis in this work are based on data reported in experimental studies using Dimethyl sulfoxide (DMSO).[26,28,29] The extraction efficiency of tetrahydrofuran (THF) is not fully understood. We have conducted sensitivity analysis considering a wide range of indigoidine extraction efficiencies (70-99%). $^{\vartheta}$Data gathered from online sources (https://www.selinawamucii.com/insights/prices/united-states-of-america/soya-sauce/ and https://www.intratec.us/chemical-markets/tetrahydrofuran-price).

S8. Impact of Host Microbes on Capital and Operating Costs

Figure S1 depicts capital and operating costs for different indigoidine biorefinery configurations using one of five microbial hosts: *E. coli*, *S. cerevisiae*, *R. toruloides*, *P. putida*, and *C. glutamicum*. The results indicate that the bioconversion stage is consistently among the top four contributors to total CapEx. For *P. putida*, which is the only organism currently shown to convert lignin-derived aromatics to indigoidine, the bioconversion stage's share of CapEx is larger than for other hosts because of the increased reactor volume needed to accommodate more carbon sources and longer residence times (Fig. S1-e & g). This highlights the advantage of microbes with a high production rate, such as *C. glutamicum* (Fig. S1-i) which also uses aromatics but can do so with a shorter residence time (Fig. S1-e & g). The total CapEx for a biorefinery using *R. toruloides* or *C. glutamicum* does not decrease as the yield increases from the current to baseline (50%) scenario because neither of these organisms have yet been shown to convert aromatics to indigoidine, so the baseline scenario includes the need for further increases in bioconversion capital requirements to accommodate aromatics, with a corresponding decrease in on-site energy generation from residual unconverted lignin. In the optimal yield scenario, both *C. glutamicum* and *S. cerevisiae* include modest CapEx required for recovery of co-products (lactic acid and ethanol, respectively), which increases their CapEx relative to host microbes without co-products.

Across all scenarios, a consistent theme is the advantage of reducing residual solids that must be burned for on-site energy generation. This has been shown to be advantageous in prior studies[33] and is also useful from the perspective of minimizing air pollutant emissions.[34,35] Another key driver of CapEx in biorefineries is the need for on-site wastewater treatment, as shown in Fig. S1. Hosts that utilize lignin-derived aromatics suffer from elevated CapEx due to the additional wastewater generated during lignin depolymerization. *E. coli* and *S. cerevisiae*, as a result, have approximately 40% lower wastewater treatment CapEx.

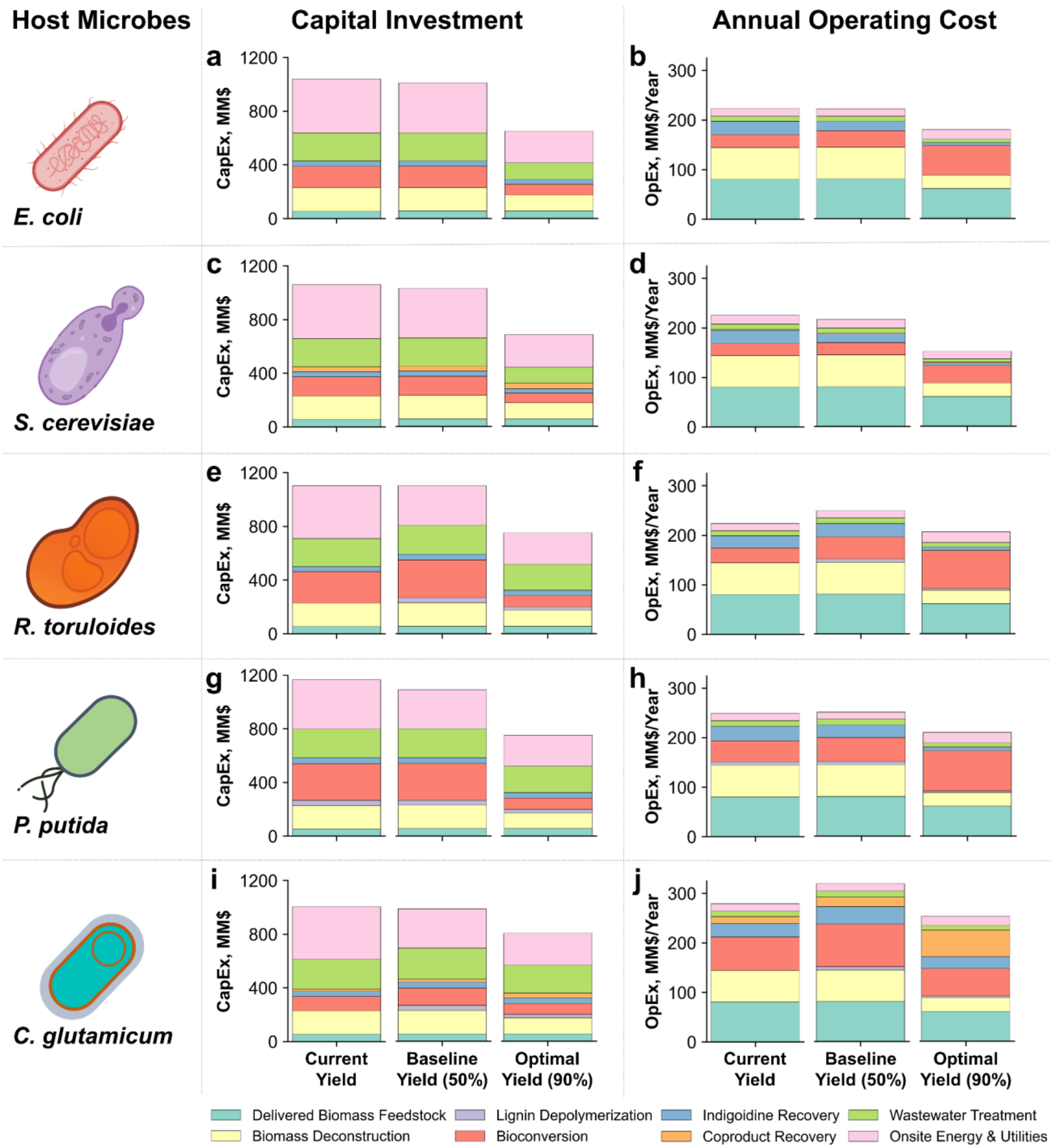

Figure S2. Capital investment (CapEx) and annual operating cost (OpEx) of indigoidine biorefinery scenarios for each host microbe. In this figure, a, c, e, g, and i represent the capital cost, while b, d, f, h, and j represent the annual operating cost of the modeled indigoidine biorefinery using the host microbes *E. coli*, *S. cerevisiae*, *R. toruloides*, *P. putida*, *and C. glutamicum*, respectively. The current yield scenario reflects yields demonstrated at the bench scale. The baseline scenario assumes 50% of the maximum theoretical yield. The optimal scenario targets 90% of the theoretical yield and incorporates system-level enhancements, including a 90% sugar yield from biomass, a 48-hour bioconversion time, 25 wt% solid loading for bioconversion, and a low-lignin (10 wt%), high-carbohydrate (70 wt%) feedstock.

The choice of microbial host, and whether it utilizes aromatics, also has a substantial impact on annual operating cost (OpEx), as shown in Fig. S1 b, d, f, h, and j. Across most scenarios and hosts, delivered feedstock remains the single largest component of the OpEx. In both the current and baseline scenarios, the feedstock cost remains unchanged. In the optimal scenario, delivered feedstock costs are reduced to a practical minimum (see Table 1). *E. coli* and *S. cerevisiae* have the lowest OpEx totals across all scenarios, in large part because they do not utilize aromatics. For both *C. glutamicum* and *R. toruloides*, the OpEx rises in the baseline case relative to the current case because only the baseline case begins to introduce lignin depolymerization and conversion of the resulting aromatics. The high OpEx for *C. glutamicum* may appear surprising. This is driven by two primary factors: the high cost of lactic acid co-product recovery (1.7 kg produced per kg of indigoidine) and the additional cost of soy sauce,[30] which improved the intracellular supply of glutamine (an indigoidine precursor) and enhanced the titer.[30] Modern lactic acid recovery techniques, such as solvent- and membrane-based processes,[15] could help reduce the recovery cost. These methods either reduce or eliminate disposal cost of waste gypsum and lactic acid purification cost and energy.

Increasing carbon source content in the hydrolysate in the optimal scenario increases the OpEx for the bioconversion stage for most microbes except *C. glutamicum*. For *P. putida* and *R. toruloides*, the bioconversion stage becomes the single largest contributor to OpEx as these microbes require relatively more nitrogen sources, among others (Table 1). In contrast, the bioconversion stage OpEx for *C. glutamicum* is lower in the optimal scenario compared to the current and baseline scenarios because the reduction in inoculum loading from 10% to 5% in the optimal scenario also reduces the total amount of expensive soy sauce required for *C. glutamicum* growth. Such a reduction in inoculum loading is less impactful for other microbes, which are grown in minimal media. [26–29]

S9. Enhancing Economic and Environmental Benefits of Microbial Indigoidine

The results indicate that while improving microbial performance is important, more significant reductions can be achieved through system-level improvements, as microbial enhancements alone are not sufficient to reach higher titers, rates, and yields.

Tailored plant engineering efforts are essential to optimize biomass composition based on downstream conversion pathways and biorefinery configurations. When lignin is not fully utilized, it can serve as a valuable source of energy for generating onsite heat and electricity. However, burning lignin in its moist form (around 20% moisture) in a biorefinery is inefficient, which can unnecessarily increase capital costs. Whether lignin is utilized or not, fine-tuning biomass composition through system-level analysis is beneficial (Figs.S2 & S3), as it can impact capital costs, material and energy requirements, and the need for external energy sourcing. If lignin is insufficient to generate onsite heat and electricity, the biorefinery may only produce process steam, with the necessary electricity being sourced directly from the grid. As solar and wind energy are likely to constitute a larger share of grid electricity in the future, the cost and

carbon footprint of electricity generated from moist lignin at the biorefinery may become less competitive.

In addition to reducing capital and operating costs, fine-tuning biomass composition—such as increasing the carbohydrate fraction by reducing lignin, as considered in the optimal yield scenario—raises the concentration of sugars entering the bioreactor, thereby boosting the titer and productivity of indigoidine without further improvements in biomass deconstruction. Further enhancing the efficiency of biomass deconstruction can elevate sugar concentrations (Figs.S2 & S3) even more, improving titer and productivity without necessarily increasing product yield.

Higher solid loadings in biomass deconstruction have the potential to enhance bioproduct titer and productivity by increasing the concentration of sugars, lignin monomers, or both. A significant challenge in lignocellulosic biorefineries is the typically low concentration of resulting sugars or carbon sources. Increasing solid loadings during biomass deconstruction enables higher carbon source concentrations without the need for additional capital and operating costs associated with sugar concentration units. In either case, increasing the concentration of carbon sources is crucial for achieving greater economic benefits and reducing GHG emissions (Figs.S2 & S3).

While higher concentrations of carbon sources can significantly increase the titer and rates of bioproducts, a major challenge for microbial bioconversion is whether microbes can efficiently catabolize such concentrated carbon sources (Figs. S2 & S3). Experimental sugar or aromatic concentrations are often 18 times lower than the model concentrations expected in optimal future scenarios. Addressing this gap is crucial, particularly in determining which microbes can utilize high concentrations of carbon sources while still achieving similar yields with the same productivity. Additionally, it is essential to identify which microbes are sensitive to changes in sugar or lignin monomer concentration. This highlights the importance of exploring or engineering microbes that can maintain high performance even as input parameters fluctuate, requiring minimal amounts of oxygen, nitrogen sources, and other specific media parameters for growth and function. While utilizing a high sugar concentration or achieving high titer and productivity is less challenging in the case of indigoidine, this is particularly due to the higher market value of the incumbent product, synthetic indigo dye. However, it is very challenging to achieve a selling price below $1/kg. This challenge is similar to that faced by many biofuels. The results underscore the importance of focusing on high-value products if achieving a larger titer and rate is impossible.

To maximize economic benefits, selecting microbes capable of efficiently catabolizing all available carbon sources is crucial. However, if lignin is limited, as considered in the optimal yield scenario, focusing solely on maximizing carbohydrate utilization could yield similar results. For instance, the minimum selling prices of indigoidine produced in *E. coli* and *S. cerevisiae* using only sugars are comparable to those in *P. putida* and *R. toruloides*, which utilize both sugars and aromatics. While lignin-derived aromatics can enhance product yields, they also demand more energy and materials, particularly nitrogen sources, for catabolism. This

highlights the importance of field-to-product system analysis to fully optimize the bioconversion process for the selected host microbes.

While many natural microbes have been engineered to maximize indigoidine production by suppressing coproduct pathways, this approach often requires substantial resources, time, and effort. Some pathways, such as those producing ethanol and lactic acid, require less energy, while others, like indigoidine production, demand higher energy and materials. A shift in strain engineering efforts is needed—particularly in reassessing the conventional focus on maximizing a single product while suppressing coproduct pathways—to achieve greater economic and GHG emissions reduction benefits with minimal engineering efforts.

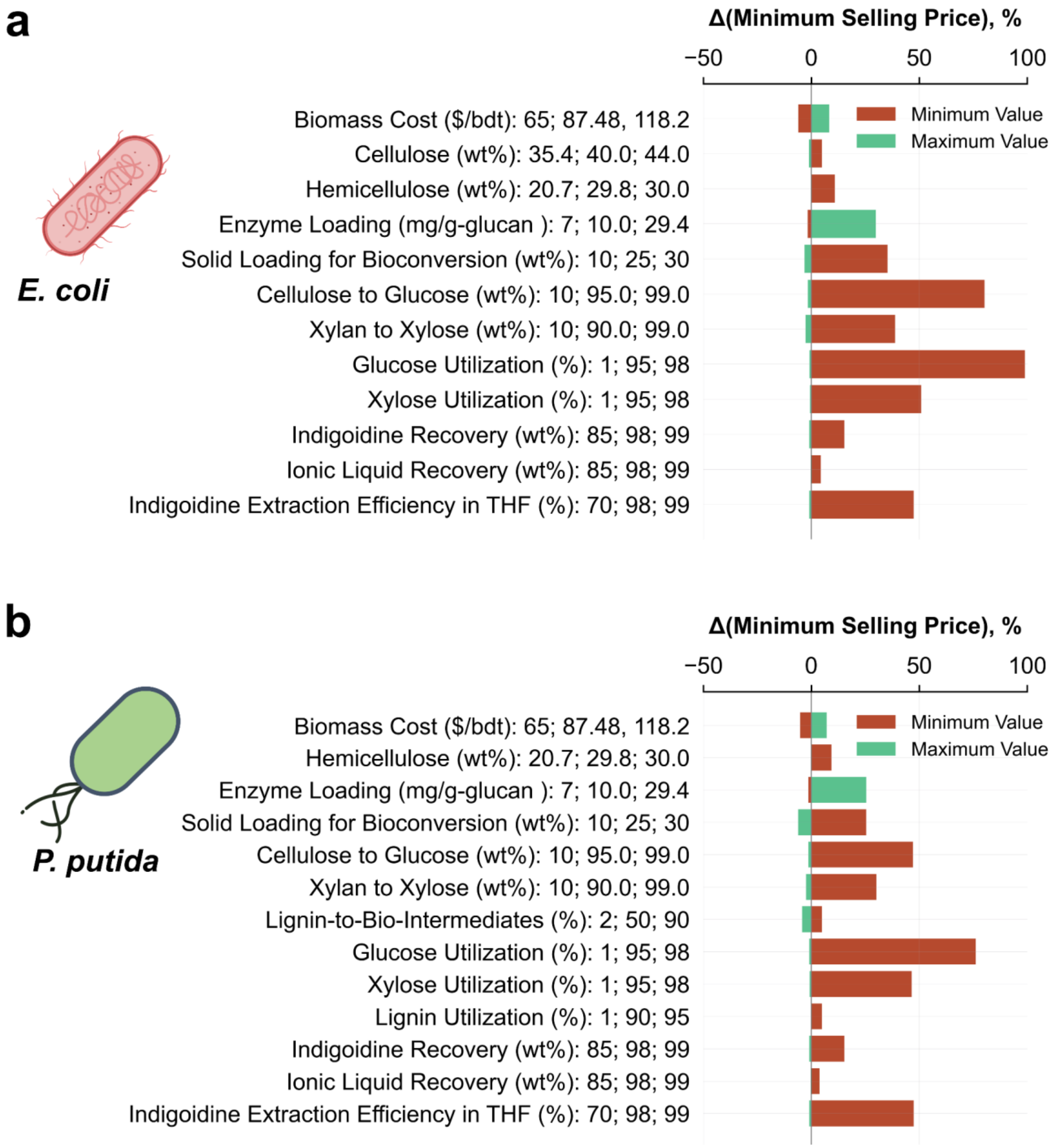

Figure S3. Most influential parameters affecting the minimum selling price of indigoidine. In this figure, a and b represent the sensitivity in the minimum selling price of indigoidine produced in *E. coli* and *P. putida*, respectively. We consider two representative microbes, *E. coli*, which utilizes only sugars, and *P. putida*, which utilizes both sugars and aromatics. In this figure, bdt stands for bone-dry metric ton, and THF stands for tetrahydrofuran.

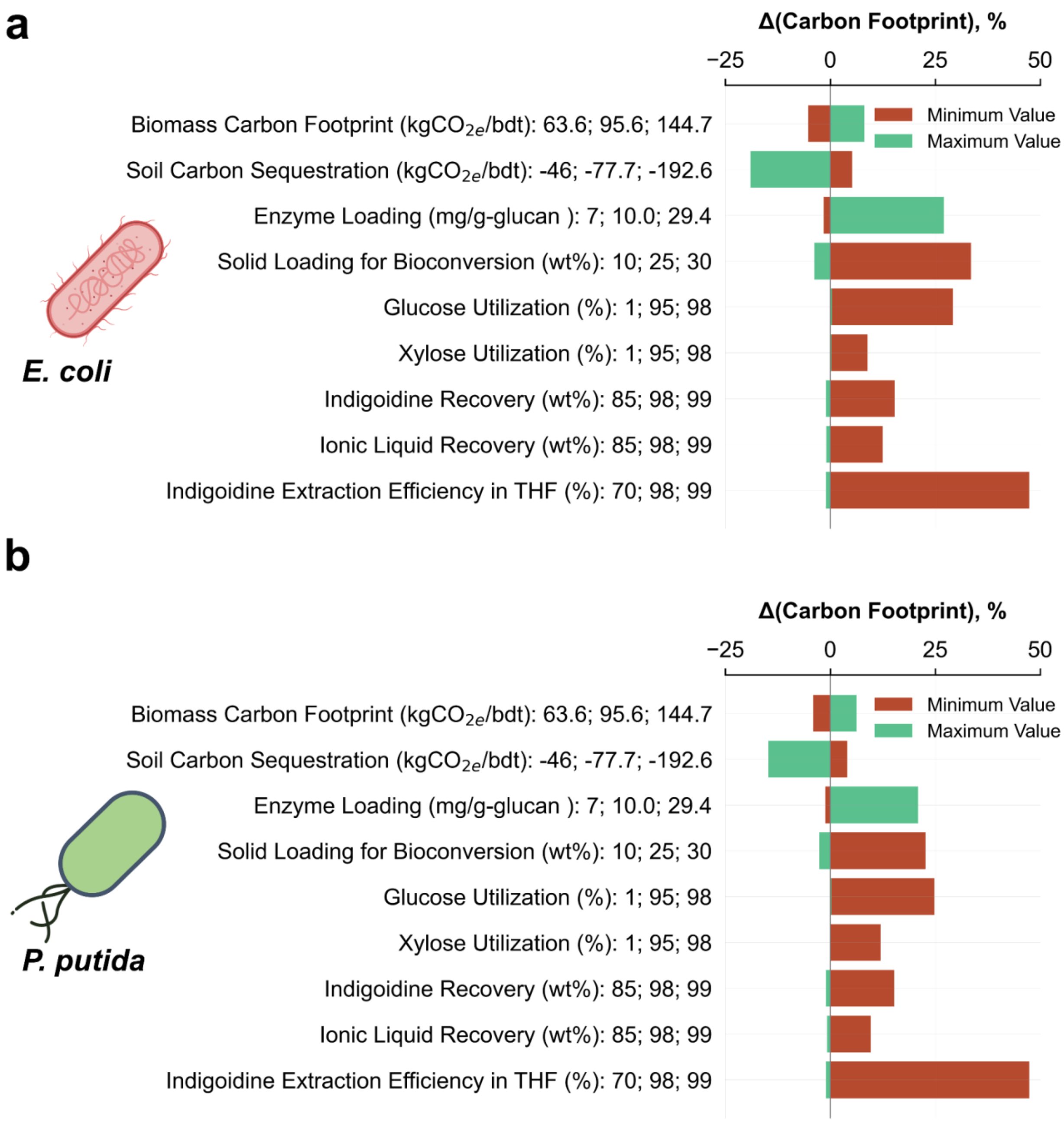

Figure S4. Most influential parameters affecting the carbon footprint of indigoidine. In this figure, a and b represent the sensitivity in the greenhouse gas emissions of indigoidine produced in *E. coli* and *P. putida*, respectively. We consider two representative microbes, *E. coli*, which utilizes only sugars, and *P. putida*, which utilizes both sugars and aromatics. In this figure, bdt stands for bone-dry metric ton, and THF stands for tetrahydrofuran.

Table S4. Major process equipment sizes, purchase prices and installed costs

Equipment Number	Equipment Name	Equipment Size	Unit	Installation Factor	Quantity	Unit Rate ($)	Purchasing Price ($)	Installed Cost
GBX-109	Truck Scale	52083.33	kg/h	0.7	2	121,000	242,000	411,400
PM-101	Centrifugal Pump	2.8	kW	1.3	1	37,000	37,000	85,100
P-224	Centrifugal Pump	4.31	kW	1.3	1	45,000	45,000	103,500
R-101	Pretreatment Reactor	93.89	m^3	0.5	1	1,079,000	1,079,000	1,618,500
P-602	Centrifugal Pump	2.86	kW	1.3	1	38,000	38,000	87,400
P-606	Centrifugal Pump	2.66	kW	1.3	1	37,000	37,000	85,100
CT-608	Clarifier	620.06	m^2	0.5	1	9,546,000	9,546,000	14,319,000
BT-605	Blending Tank	0.12	m^3	1.5	1	13,000	13,000	32,500
P-611	Centrifugal Pump	4.52	kW	1.3	1	46,000	46,000	105,800
ST-507	Flat Bottom Tank	2913.67	m^3	0.51	2	419,000	838,000	1,265,380
P-616	Centrifugal Pump	2.81	kW	1.3	1	37,000	37,000	85,100
HX-102	Heat Exchanger	79.07	m^2	1.2	2	137,000	274,000	602,800
M-102	Centrifugal Fan	183664	m^3/h	1	1	59,000	59,000	118,000
PM-116	Centrifugal Pump	130.06	kW	1.3	1	187,000	187,000	430,100
CSP-106	Component Splitter	528.83	MT/h	0.61	1	4,280,000	4,280,000	6,890,800
RT-606	Anaerobic Digester	268.62	MT/h	0.11	2	33,000,000	66,000,000	73,260,000
RT-607	Aerobic Bio-Oxydation	1088.28	MT/h	1.07	1	4,025,000	4,025,000	8,331,750
SG-101	Steam Generator	232.16	MT/h	0.8	1	41,103,000	41,103,000	73,985,400
T-101	Turbine-Generator	34.55	MW	0.8	1	12,621,000	12,621,000	22,717,800
PM-114	Centrifugal Pump	96.4	kW	1.3	1	166,000	166,000	381,800
HX-103	Heat Exchanger	81.87	m^2	1.2	4	140,000	560,000	1,232,000
G-101	Compressor	76.32	kW	0.6	1	118,000	118,000	188,800
V-105	Blending Tank	74977.58	L	1.5	12	425,000	5,100,000	12,750,000
PM-118	Centrifugal Pump	1.85	kW	1.3	1	31,000	31,000	71,300
HX-105	Heat Exchanger	2.51	m^2	1.2	1	17,000	17,000	37,400
PM-123	Centrifugal Pump	94.91	kW	1.3	1	165,000	165,000	379,500
PM-115	Centrifugal Pump	163.97	kW	0.5	2	205,000	410,000	615,000
CSP-104	Component Splitter	47.55	MT/h	0.59	1	816,000	816,000	1,297,440
ST-207	Receiver Tank	126.19	m^3	1.6	1	89,000	89,000	231,400
SR-102	Seed Reactor	1.75	m^3	0.5	1	734,000	734,000	1,101,000
SR-104	Seed Reactor	3483	m^3	0.5	5	2,106,000	10,530,000	15,795,000
PM-103	Centrifugal Pump	0.01	kW	2.1	1	12,000	12,000	37,200
PM-104	Centrifugal Pump	4.31	kW	1.3	1	45,000	45,000	103,500
PM-106	Centrifugal Pump	7.17	kW	2.8	1	56,000	56,000	212,800
BC-104	Belt Conveyor	19.81	m	0.7	1	1,743,000	1,743,000	2,963,100
HX-108	Heat Exchanger	17.79	m^2	1.2	1	56,000	56,000	123,200
HX-110	Heat Exchanger	172.2	m^2	1.2	7	66,000	462,000	1,016,400
UF-103	Ultrafilter	76.6	m^2	0.5	23	173,000	3,979,000	5,968,500
PM-151	Centrifugal Pump	13.98	kW	1.3	1	77,000	77,000	177,100

Contd….

Equipment Number	Equipment Name	Equipment Size	Unit	Installation Factor	Quantity	Unit Rate ($)	Purchasing Price ($)	Installed Cost
PM-152	Centrifugal Pump	0.07	kW	1.3	1	12,000	12,000	27,600
PM-153	Centrifugal Pump	6.91	kW	2.8	1	113,000	113,000	429,400
CSP-111	Pervaporation Unit	358.67	MT/h	0.5	1	14,860,000	14,860,000	22,290,000
HX-112	Heat Exchanger	25.72	m^2	0.5	1	70,000	70,000	105,000
PM-150	Centrifugal Pump	1.47	kW	1.3	1	28,000	28,000	64,400
PM-134	Centrifugal Pump	2.21	kW	1.3	1	34,000	34,000	78,200
V-110	Flat Bottom Tank	1801.71	m3	0.7	1	725,000	725,000	1,232,500
HX-106	Heat Exchanger	196.72	m^2	1.2	23	73,000	1,679,000	3,693,800
GBX-105	Truck Dumper	52083.33	kg/h	0.7	2	531,000	1,062,000	1,805,400
GBX-106	Truck Dumper Hopper	52083.33	kg/h	0.7	2	551,000	1,102,000	1,873,400
SR-103	Shredder	6.94	MT/h	0.5	15	132,000	1,980,000	2,970,000
VSCR-101	Vibrating Screen	104166.67	kg/h	0.5	1	63,000	63,000	94,500
GBX-112	Dust Collection Unit	5687.92	kg/h	0.7	1	81,000	81,000	137,700
BC-101	Belt Conveyor	19.81	m	0.7	1	4,240,000	4,240,000	7,208,000
SL-101	Silo	22875.82	m^3	0.7	1	2,162,000	2,162,000	3,675,400
BC-102	Belt Conveyor	0.76	m	0.7	3	17,000	51,000	86,700
BC-103	Belt Conveyor	19.81	m	0.7	2	1,743,000	3,486,000	5,926,200
SR-101	Shredder	6.32	MT/h	0.5	9	129,000	1,161,000	1,741,500
BC-108	Belt Conveyor	19.81	m	0.7	1	1,743,000	1,743,000	2,963,100
BC-109	Belt Conveyor	19.81	m	0.7	1	1,743,000	1,743,000	2,963,100
PM-137	Centrifugal Pump	2.76	kW	1.3	1	76,000	76,000	174,800
SC-101	Screw Conveyor	7.62	m	0.7	5	27,000	135,000	229,500
V-114	Blending Tank	58467.73	L	1.5	1	128,000	128,000	320,000
PM-138	Centrifugal Pump	0.57	kW	1.3	1	39,000	39,000	89,700
BC-106	Belt Conveyor	19.81	m	0.7	1	4,240,000	4,240,000	7,208,000
PM-140	Centrifugal Pump	0.61	kW	1.3	1	40,000	40,000	92,000
C-105	Distillation Column	16993.72	L	1	2	386,000	772,000	1,544,000
PM-141	Centrifugal Pump	0.24	kW	1.3	1	27,000	27,000	62,100
SC-102	Screw Conveyor	7.62	m	0.7	4	27,000	108,000	183,600
TFE-101	Thin Film Evaporator	40.35	m^2	1	1	188,000	188,000	376,000
M-105	Centrifugal Fan	23184	m^3/h	0.09	1	136,000	136,000	148,240
V-104	Flat Bottom Tank	959.85	m^3	0.7	3	395,000	1,185,000	2,014,500
CT-101	Cooling Tower	81119.53	L	0.5	10	78,000	780,000	1,170,000
PM-124	Centrifugal Pump	158.27	kW	2.1	2	50,000	100,000	310,000
GBX-102	Chilled Water Unit	586652.7	kg/h	0.6	4	992,000	3,968,000	6,348,800
BC-110	Belt Conveyor	19.81	m	0.7	1	1,743,000	1,743,000	2,963,100
R-102	Stirred Reactor	8.51	m^3	0.7	1	4,976,000	4,976,000	8,459,200

Contd….

Table S4. Contd.

Equipment Number	Equipment Name	Equipment Size	Unit	Installation Factor	Quantity	Unit Rate ($)	Purchasing Price ($)	Installed Cost
PM-122	Centrifugal Pump	0.33	kW	1.3	1	15,000	15,000	34,500
PM-125	Centrifugal Pump	0.64	kW	1.3	1	20,000	20,000	46,000
PM-127	Centrifugal Pump	0.34	kW	1.3	1	15,000	15,000	34,500
M-101	Centrifugal Fan	763407.34	L/h	0.09	1	25,000	25,000	27,250
HX-101	Heat Exchanger	8.24	m^2	0.5	1	35,000	35,000	52,500
PM-131	Centrifugal Pump	0.65	kW	1.3	1	20,000	20,000	46,000
PM-109	Centrifugal Pump	0.66	kW	1.3	1	20,000	20,000	46,000
V-117	Neutralizer	18579.69	L	1	1	104,000	104,000	208,000
GBX-101	Rotary Vacuum Filter	338158.15	kg/h	0.5	1	3,198,000	3,198,000	4,797,000
GBX-108	Rotary Vacuum Filter	442959.76	kg/h	0.5	1	4,189,000	4,189,000	6,283,500
GBX-110	Rotary Vacuum Filter	117506.87	kg/h	0.5	1	1,111,000	1,111,000	1,666,500
GBX-111	Rotary Vacuum Filter	19289.08	kg/h	0.5	1	182,000	182,000	273,000
FR-103	Bubble Column Bioreactor	997.97	m^3	0.5	27	909,000	24,543,000	36,814,500
V-108	Blending Tank	124.5	m^3	1.5	1	118,000	118,000	295,000
M-104	Centrifugal Fan	64602	m^3/h	1	1	27,000	27,000	54,000
V-101	Flat Bottom Tank	83.44	m^3	0.7	1	98,000	98,000	166,600
M-103	Centrifugal Fan	42896	m^3/h	1	1	20,000	20,000	40,000

References

(1) Linke, J. A.; Rayat, A.; Ward, J. M. Production of indigo by recombinant bacteria. *Bioresour. Bioprocess.* **2023**, *10*, 20.

(2) Periyasamy, A. P.; Militky, J. Denim processing and health hazards. In *Sustainability in Denim*; Elsevier, **2017**; pp. 161–196.

(3) Wild Colours. Why dye with Indigo, **2024**, https://www.wildcolours.co.uk/html/why_indigo.html#:~:text=Indigo%20is%20very%20concentrated%3A%2010,chemicals%20in%20a%20fermentation%20vat.&text=Overdye%20a%20woad%20or%20indigo,such%20as%20weld%20or%20fustic. (accessed Sep 5, 2024).

(4) Bidart, G. N.; Teze, D.; Jansen, C. U.; Pasutto, E.; Putkaradze, N.; Sesay, A.-M.; Fredslund, F.; Lo Leggio, L.; Ögmundarson, O.; Sukumara, S.; Qvortrup, K. Chemoenzymatic indican for light-driven denim dyeing. *Nat. Commun.* **2024**, *15*, 1489.

(5) Argonne National Laboratory. GREET; Argonne National Laboratory, Argonne, IL, **2023**, https://greet.es.anl.gov/ (accessed Sep 06, 2023).

(6) DENIMSANDJEANS. Sense the Substance of BIO INDIGO, **2020**, https://www.denimsandjeans.com/environment/sense-the-substance-of-bio-indigo/42439 (accessed Sep 5, 2024)..

(7) Ellen MacArthur Foundation. A new textiles economy: Redesigning fashion’s future, **2017**, https://emf.thirdlight.com/file/24/uiwtaHvud8YIG_uiSTauTlJH74/A%20New%20Textiles%20Economy%3A%20Redesigning%20fashion%E2%80%99s%20future.pdf (accessed Dec 31, 2019).

(8) QUANTIS. Environmental Impact of the Global Apparel and Footwear Industries Study, **2018**,https://quantis-intl.com/wp-content/uploads/2018/03/measuringfashion_globalimpactstudy_full-report_quantis_cwf_2018a.pdf (accessed Dec 31, 2019).

(9) Karthik, T.; Murugan, R. Carbon footprint in denim manufacturing. In *Sustainability In Denim*; Muthu, S. S., Ed.; Woodhead Publishing: Duxford, **2017**; pp. 125–159.

(10) Baral, N. R.; Dahlberg, J.; Putnam, D.; Mortimer, J. C.; Scown, C. D. Supply cost and life-cycle greenhouse gas footprint of dry and ensiled biomass sorghum for biofuel production. *ACS Sustain. Chem. Eng.* **2020**, *8*, 15855–15864.

(11) Huntington, T.; Baral, N. R.; Yang, M.; Sundstrom, E.; Scown, C. D. Machine learning for surrogate process models of bioproduction pathways. *Bioresour. Technol.* **2023**, *370*, 128528.

(12) Baral, N. R.; Davis, R.; Bradley, T. H. Supply and value chain analysis of mixed biomass feedstock supply system for lignocellulosic sugar production. *Biofuels, Bioprod. Bioref.* **2019**, *13*, 635–659.

(13) Roni, M. S.; Thompson, D.; Hartley, D.; Searcy, E.; Nguyen, Q. Optimal blending management of biomass resources used for biochemical conversion. *Biofuels, Bioprod. Bioref.* **2018**, *12*, 624–648.

(14) Humbird, D.; Davis, R.; Tao, L.; Kinchin, C.; Hsu, D.; Aden, A.; Schoen, P.; Lukas, J.; Olthof, B.; Worley, M.; Sexton, D. Process Design and Economics for Biochemical Conversion of Lignocellulosic Biomass to Ethanol: Dilute-Acid Pretreatment and

Enzymatic Hydrolysis of Corn Stover; National Renewable Energy Laboratory (NREL), Golden, CO (United States), **2011**, https://www.nrel.gov/docs/fy11osti/47764.pdf (accessed Sep 16, 2022).
(15) Li, C.; Gao, M.; Zhu, W.; Wang, N.; Ma, X.; Wu, C.; Wang, Q. Recent advances in the separation and purification of lactic acid from fermentation broth. *Process Biochemistry* **2021**, *104*, 142–151.
(16) Lan, K.; Xu, S.; Li, J.; Hu, C. Recovery of Lactic Acid from Corn Stover Hemicellulose-Derived Liquor. *ACS Omega* **2019**, *4*, 10571–10579.
(17) Komesu, A., Maciel, M. W., & Maciel Filho, R. Separation and purification technologies for lactic acid–A brief review. BioResources, **2017**, 12(3), 6885-6901.
(18) Marchesan, A. N.; Leal Silva, J. F.; Maciel Filho, R.; Wolf Maciel, M. R. Techno-Economic Analysis of Alternative Designs for Low-pH Lactic Acid Production. *ACS Sustain. Chem. Eng.* **2021**, *9*, 12120–12131.
(19) Baral, N. R.; Kavvada, O.; Mendez-Perez, D.; Mukhopadhyay, A.; Lee, T. S.; Simmons, B. A.; Scown, C. D. Techno-economic analysis and life-cycle greenhouse gas mitigation cost of five routes to bio-jet fuel blendstocks. *Energy Environ. Sci.* **2019**, *12*, 807–824.
(20) Davis, R. E.; Grundl, N. J.; Tao, L.; Biddy, M. J.; Tan, E. C.; Beckham, G. T.; Humbird, D.; Thompson, D. N.; Roni, M. S. Process design and economics for the conversion of lignocellulosic biomass to hydrocarbon fuels and coproducts: 2018 biochemical design case update; biochemical deconstruction and conversion of biomass to fuels and products via integrated biorefinery pathways; National Renewable Energy Laboratory (NREL), Golden, CO (United States), **2018**, https://www.nrel.gov/docs/fy19osti/71949.pdf (accessed Sep 16, 2022).
(21) Orth, J. D.; Thiele, I.; Palsson, B. Ø. What is flux balance analysis? *Nat. Biotechnol.* **2010**, *28*, 245–248.
(22) Heirendt, L.; Arreckx, S.; Pfau, T.; Mendoza, S. N.; Richelle, A.; Heinken, A.; Haraldsdóttir, H. S.; Wachowiak, J.; Keating, S. M.; Vlasov, V.; Magnusdóttir, S. Creation and analysis of biochemical constraint-based models using the COBRA Toolbox v.3.0. *Nat. Protoc.* **2019**, *14*, 639–702.
(23) Gautam, S.; Baral, N. R.; Mishra, U.; Scown, C. D. Impact of bioenergy feedstock carbon farming on sustainable aviation fuel viability in the United States. *Proc Natl Acad Sci USA* **2023**, *120*, e2312667120.
(24) Magurudeniya, H. D.; Baral, N. R.; Rodriguez, A.; Scown, C. D.; Dahlberg, J.; Putnam, D.; George, A.; Simmons, B. A.; Gladden, J. M. Use of ensiled biomass sorghum increases ionic liquid pretreatment efficiency and reduces biofuel production cost and carbon footprint. Green Chem. **2021**, 23, 3127–3140.
(25) Baral, N. R.; Kavvada, O.; Mendez Perez, D.; Mukhopadhyay, A.; Lee, T. S.; Simmons, B. A.; Scown, C. D. Greenhouse gas footprint, water-intensity, and production cost of bio-based isopentenol as a renewable transportation fuel. *ACS Sustain. Chem. Eng.* **2019**, *7*, 15434–15444.
(26) Xu, F.; Gage, D.; Zhan, J. Efficient production of indigoidine in *Escherichia coli*. *J. Ind. Microbiol. Biotechnol.* **2015**, *42*, 1149–1155.
(27) Wehrs, M.; Prahl, J.-P.; Moon, J.; Li, Y.; Tanjore, D.; Keasling, J. D.; Pray, T.; Mukhopadhyay, A. Production efficiency of the bacterial non-ribosomal peptide

indigoidine relies on the respiratory metabolic state in *S. cerevisiae*. *Microb. Cell Fact.* **2018**, *17*, 193.
(28) Wehrs, M.; Gladden, J. M.; Liu, Y.; Platz, L.; Prahl, J.-P.; Moon, J.; Papa, G.; Sundstrom, E.; Geiselman, G. M.; Tanjore, D.; Keasling, J.D. Sustainable bioproduction of the blue pigment indigoidine: Expanding the range of heterologous products in *R. toruloides* to include non-ribosomal peptides. *Green Chem.* **2019**, *21*, 3394–3406.
(29) Banerjee, D.; Eng, T.; Lau, A. K.; Sasaki, Y.; Wang, B.; Chen, Y.; Prahl, J.-P.; Singan, V. R.; Herbert, R. A.; Liu, Y.; Tanjore, D. Genome-scale metabolic rewiring improves titers rates and yields of the non-native product indigoidine at scale. *Nat. Commun.* **2020**, *11*, 5385.
(30) Ghiffary, M. R.; Prabowo, C. P. S.; Sharma, K.; Yan, Y.; Lee, S. Y.; Kim, H. U. High-Level Production of the Natural Blue Pigment Indigoidine from Metabolically Engineered *Corynebacterium glutamicum* for Sustainable Fabric Dyes. *ACS Sustain. Chem. Eng.* **2021**, *9*, 6613–6622.
(31) Schutyser, W., Renders, A. T., Van den Bosch, S., Koelewijn, S. F., Beckham, G. T., Sels, B. F. Chemicals from lignin: an interplay of lignocellulose fractionation, depolymerisation, and upgrading. *Chem. Soc. Rev.* **2018**, 47(3), 852-908.
(32) Choudhary, H., Das, L., Pelton, J. G., Sheps, L., Simmons, B. A., Gladden, J. M., Singh, S. Funneled Depolymerization of Ionic Liquid-Based Biorefinery “Heterogeneous” Lignin into Guaiacols over Reusable Palladium Catalyst. *Chem. Eur. J.* **2023**, 29(27), e202300330.
(33) Scown, C. D.; Gokhale, A. A.; Willems, P. A.; Horvath, A.; McKone, T. E. Role of lignin in reducing life-cycle carbon emissions, water use, and cost for United States cellulosic biofuels. *Environ. Sci. Technol.* **2014**, *48*, 8446–8455.
(34) Eberle, A.; Bhatt, A.; Zhang, Y.; Heath, G. Potential air pollutant emissions and permitting classifications for two biorefinery process designs in the united states. *Environ. Sci. Technol.* **2017**, *51*, 5879–5888.
(35) Bhatt, A.; Zhang, Y.; Davis, R.; Eberle, A.; Heath, G. Economic implications of incorporating emission controls to mitigate air pollutants emitted from a modeled hydrocarbon-fuel biorefinery in the United States. *Biofuels, Bioprod. Bioref.* **2016**, *10*, 603–622.
